# Maternal Oct-4 is a potential key regulator of the developmental competence of mouse oocytes

**DOI:** 10.1186/1471-213X-8-97

**Published:** 2008-10-06

**Authors:** Maurizio Zuccotti, Valeria Merico, Lucia Sacchi, Michele Bellone, Thore C Brink, Riccardo Bellazzi, Mario Stefanelli, Carlo Alberto Redi, Silvia Garagna, James Adjaye

**Affiliations:** 1 Sezione di Istologia ed Embriologia, Dipartimento di Medicina Sperimentale, Universita' degli Studi di Parma, Parma, Italy; 2 Laboratorio di Biologia dello Sviluppo, Dipartimento di Biologia Animale, Universita' degli Studi di Pavia, Pavia, Italy; 3 Dipartimento di Informatica e Sistemistica, Universita' degli Studi di Pavia, Pavia, Italy; 4 Molecular Embryology and Aging Group, Max-Planck Institute for Molecular Genetics, Berlin, Germany; 5 Fondazione IRCCS, Policlinico San Matteo, Pavia, Italy

## Abstract

**Background:**

The maternal contribution of transcripts and proteins supplied to the zygote is crucial for the progression from a gametic to an embryonic control of preimplantation development. Here we compared the transcriptional profiles of two types of mouse MII oocytes, one which is developmentally competent (MII^SN ^oocyte), the other that ceases development at the 2-cell stage (MII^NSN ^oocyte), with the aim of identifying genes and gene expression networks whose misregulated expression would contribute to a reduced developmental competence.

**Results:**

We report that: 1) the transcription factor Oct-4 is absent in MII^NSN ^oocytes, accounting for 2) the down-regulation of Stella, a maternal-effect factor required for the oocyte-to-embryo transition and of which Oct-4 is a positive regulator; 3) eighteen Oct-4-regulated genes are up-regulated in MII^NSN ^oocytes and are part of gene expression networks implicated in the activation of adverse biochemical pathways such as oxidative phosphorylation, mitochondrial dysfunction and apoptosis.

**Conclusion:**

The down-regulation of Oct-4 plays a crucial function in a sequence of molecular processes that leads to the developmental arrest of MII^NSN ^oocytes. The use of a model study in which the MII oocyte ceases development consistently at the 2-cell stage has allowed to attribute a role to the maternal Oct-4 that has never been described before. Oct-4 emerges as a key regulator of the molecular events that govern the establishment of the developmental competence of mouse oocytes.

## Background

The early stages of mammalian development are sustained by the presence of transcripts and proteins that have been produced and stored in the oocyte during folliculogenesis. This supply is used by the morula stage in sheep and rabbit preimplantation embryos; the 4- to 8-cell stage in humans and the 2-cell stage in the mouse, at which time zygotic gene activation (ZGA) occurs and novel transcripts and proteins are expressed by the embryonic genome [reviewed in [1,2]]. What is the exact maternal contribution during this period of transition from a maternal to an embryonic control of development, and how the maternal legacy of transcripts and proteins is orchestrated through the first mitotic divisions prior to ZGA, remains unclear. An altered maternal contribution to a correct expression of zygotic genes leads to a developmental block at the time of ZGA [1,2].

This developmental block is a feature of a group of fully grown antral oocytes present in the mammalian ovary. Based on their chromatin organisation, antral oocytes can be classified in SN (surrounded nucleolus) oocytes, with a ring of chromatin surrounding the nucleolus and in NSN (not surrounded nucleolus) oocytes, with a more dispersed chromatin not surrounding the nucleolus [reviewed [3]]. These two types of gametes are present in the ovaries of all the mammalian species studied including rat [4], monkey [5], pig [6], mouse [7] and human [8]. They have been extensively studied in the mouse and a number of morphological and functional differences have been described. For example, in SN oocytes most of the centromeres localise around the compact nucleolus, whereas in NSN oocytes only the centromeres of chromosomes carrying the nucleolus organising regions are associated with the nucleolus [9,10]. Microtubule organising centres form around the nucleus of SN oocytes, but not around that of NSN oocytes [11,12]. These morphological differences have a biological relevance as they have been correlated with changes in transcription [13-15]. NSN antral oocytes are transcriptionally active and synthesise all classes of RNA, whereas SN antral oocytes are transcriptionally inactive [14].

The most striking difference between the two types of oocytes is their developmental competence. When isolated from the mouse ovary, matured to metaphase II (MII) and fertilised *in vitro*, both types of oocytes (MII^NSN ^derived from antral NSN oocytes and MII^SN ^derived from SN oocytes) are capable of developing to the 2-cell stage, but only MII^SN ^oocytes are developmentally competent beyond the 2-cell stage [16,17] and capable of reaching full term development [18].

In this study we have compared the gene expression profile of developmentally incompetent MII^NSN ^oocytes with that of developmentally competent MII^SN ^oocytes, with the aim of identifying genes and gene expression networks whose misregulated expression would contribute to a reduced developmental competence. To this end, fully grown NSN and SN antral oocytes were isolated from the ovaries, cultured *in vitro *to the MII stage and their profiles of gene expression compared. We first focused our investigation on a group of maternal-effect genes whose altered expression leads to preimplantation developmental arrest. Then, using microarrays analysis and bioinformatic resources, we identified major groups of genes, gene expression networks and biochemical pathways characteristic of the developmentally incompetent MII^NSN ^oocytes.

## Results

### Stella, a maternal-effect protein required for the oocyte-to-embryo transition, is not detected in developmentally incompetent MII^NSN ^oocytes

Our study was first focused on a specific group of gene transcripts and proteins accumulated during the oocyte growth to become necessary, after fertilisation, for successful embryogenesis. These gene products and their effects are well documented in organisms such as *Drosophila melanogaster *or *Xenopus laevis *[19,20], but in recent years, increasing evidence has been produced that maternal-effect genes are crucial in pre- [21-29] and post-implantation [28,30,31] development.

We analysed the profile of expression of the following five genes, known for playing a role during the oocyte-to-embryo transition: *Zar1*, *Npm2*, *Stella *(*Dppa3*), *Smarca4 *(*Brg1*) and *Prei3 *[22,26,27,29].

**Figure 1:**
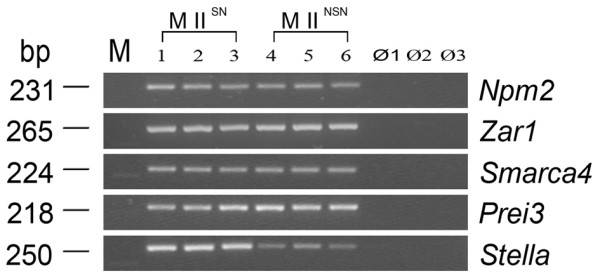
**Down-regulation of *Stella *gene expression in MII^NSN ^oocytes**. The gel electrophoresis is representative of the relative number of transcripts for each of the five maternal-effect genes analysed. When comparing MII^NSN ^with MII^SN ^oocytes, of the five genes analysed, only *Stella *was significantly regulated with a 1.4-fold down-regulation in MII^NSN ^oocytes. Samples 1–3 are three different single MII^SN ^oocytes; samples 4–6 are three different single MII^NSN ^oocytes; ∅1, RT blank; ∅2, first PCR blank; ∅3, second PCR blank; M, low mass ladder marker.

**Figure 2:**
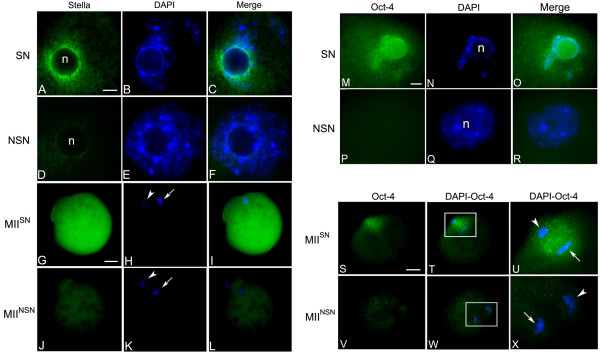
**Stella and Oct-4 proteins are not detected in antral NSN and MII^NSN ^oocytes**. (A-C) An SN antral oocyte nucleus which shows the binding of the antibody against Stella (A) around its nucleolus (n); (B) the characteristic ring of DAPI-positive chromatin surrounding the nucleolus. Bar, 3 μm. (D-F) An NSN antral oocyte nucleus negative to the antibody against Stella (D); (E) the nucleus is stained with DAPI. (G-I) A MII^SN ^oocyte that shows the binding of the antibody against Stella dispersed within the whole cytoplasm (G). Bar, 20 μm. (J-L) A MII^NSN ^oocyte negative to the antibody against Stella (J); (H, K) the arrow indicates the MII-plate, the arrowhead indicates the first polar body. (M-O) An SN antral oocyte nucleus that shows the binding of the antibody against Oct-4 localised mainly around the nucleolus. Bar, 3 μm. (P-R) An NSN antral oocyte nucleus negative to the antibody against Oct-4 (P). (S-U) A MII^SN ^oocyte that shows the binding of the antibody against Oct-4 localised around the MII-plate; (U) an enlargement of the insert shown in T. The arrow indicates the MII-plate, the arrowhead indicates the first polar body. Bar, 20 μm. (V-X) A MII^NSN ^oocyte negative to the antibody against Oct-4; (X) an enlargement of the insert shown in W.

The relative number of transcripts of the genes analysed resulted equally represented in the two types of MII oocytes, with the exception of *Stella *that was 1.4-fold down-regulated (p < 0.05) in MII^NSN ^oocytes (Figure 1). Consistently with the down-regulation of gene expression, immunostaining with an antibody against Stella did not detect the presence of the protein either in NSN antral or MII^NSN ^oocytes (Figure 2D, J), whilst in SN antral oocytes Stella co-localised around the nucleolus (Figure 2A) and in MII^SN ^oocytes was dispersed within the whole ooplasm (Figure 2G), as already shown in ovulated MII oocytes [22].

Stella down-regulation is a good candidate to explain the developmental block at the 2-cell stage that MII^NSN ^oocytes encounter following fertilisation [22]. For this reason,, we wondered what induces the down-regulation of Stella in MII^NSN ^oocytes and whether other genes could be involved in its regulation.

Stella is positively regulated by Oct-4 and Nanog based on mouse Oct-4 ChiP-pet data [32], and it is also down-regulated upon RNAi-mediated Oct-4 knockdown in human embryonic stem (ES) and embryo carcinoma (EC) cells [33,34]. A recent study has demonstrated a strong link between *Stella *and Oct-4, showing that the expression of *Stella *in mouse ES cells is regulated by Oct-4 which is necessary for maintaining a specific chromatin structure within the locus containing *Stella *[35]. The authors observed collapse of higher order chromatin structure throughout the locus and down-regulation of *Stella *expression following depletion of the *Oct-4 *gene. Additional studies using microarray analysis of gene transcription in ES cells, have described Oct-4 involvement in the regulation of chromatin modelling and chromatin-mediated transcription regulation [36].

Next in our investigation, we addressed the question whether the pattern of Oct-4 gene and protein expression in developmentally incompetent MII^NSN ^and competent MII^SN ^oocytes, in concert with the knowledge already acquired on this gene, could establish Oct-4 as a key regulator of *Stella *expression during the late phases of oocyte maturation.

### Oct-4, a chromatin-mediated regulator of *Stella *transcription, is down-regulated in developmentally incompetent MII^NSN ^oocytes

Oct-4 (Pou5f1), a nuclear transcription factor that belongs to the POU family, is involved in the regulation of the expression of a number of developmental genes and is required for the maintenance of cell pluripotency [for a review see [37]]. Oct-4 is expressed throughout folliculogenesis [38]; following fertilisation, it remains of maternal origin until the 2-cell embryo, then at the 4- to 8-cell stage is first expressed by the embryonic genome [40-42].

In an earlier study, we identified a 2-fold down-regulated expression of *Oct-4 *in NSN antral oocytes compared to SN antral oocytes [39]. In the present work, we found that down-regulation of *Oct-4 *expression in MII^NSN ^oocytes correlated with a down-regulated expression of its encoded protein as revealed by immunocytochemistry. Figure 2M–2R illustrates the immunostaining pattern of the Oct-4 protein in fully grown antral SN and NSN oocytes. While in SN oocytes the protein was intensely present and mostly localised around the nucleolus (Figure 2M), in NSN antral oocytes it was undetectable (Figure 2P). Following *in vitro *maturation to the MII phase, Oct-4 continued to be absent in MII^NSN ^oocytes (Figure 2V–2X), whereas MII^SN ^oocytes maintained the presence of the transcription factor localised around the MII plate and partially within the cytoplasm (Figure 2S–2U).

In summary, since a sound experimental evidence has demonstrated that Oct-4 regulates the expression of *Stella*, Oct-4 and Stella down-regulation in MII^NSN ^oocytes may contribute to the inability of these gametes to develop beyond the 2-cell stage. Our results suggest a novel role of maternal Oct-4 in the regulation of the developmental competence of the female gamete.

Oct-4-dependent transcriptional networks have been described regulating self-renewal and pluripotency in human [33,34,43,44] and mouse [32,36] ES and EC cells and in human mesenchymal cells [44]. These studies demonstrate that Oct-4 may interact with other genes to regulate specific biological process.

We next asked the question, is Oct-4 a key regulator of genes associated with or implicated in establishing developmental competence on oocytes? To address this, we first analysed by microarrays, global differences in gene expression between the two types of oocytes, and then searched for genes known to be regulated by Oct-4 (based on ChiP-on-chip and ChiP-Pet) in the array-derived list of regulated genes.

### Microarray-based analysis of the transcription profiles of developmentally competent and incompetent MII oocytes

**Figure 3:**
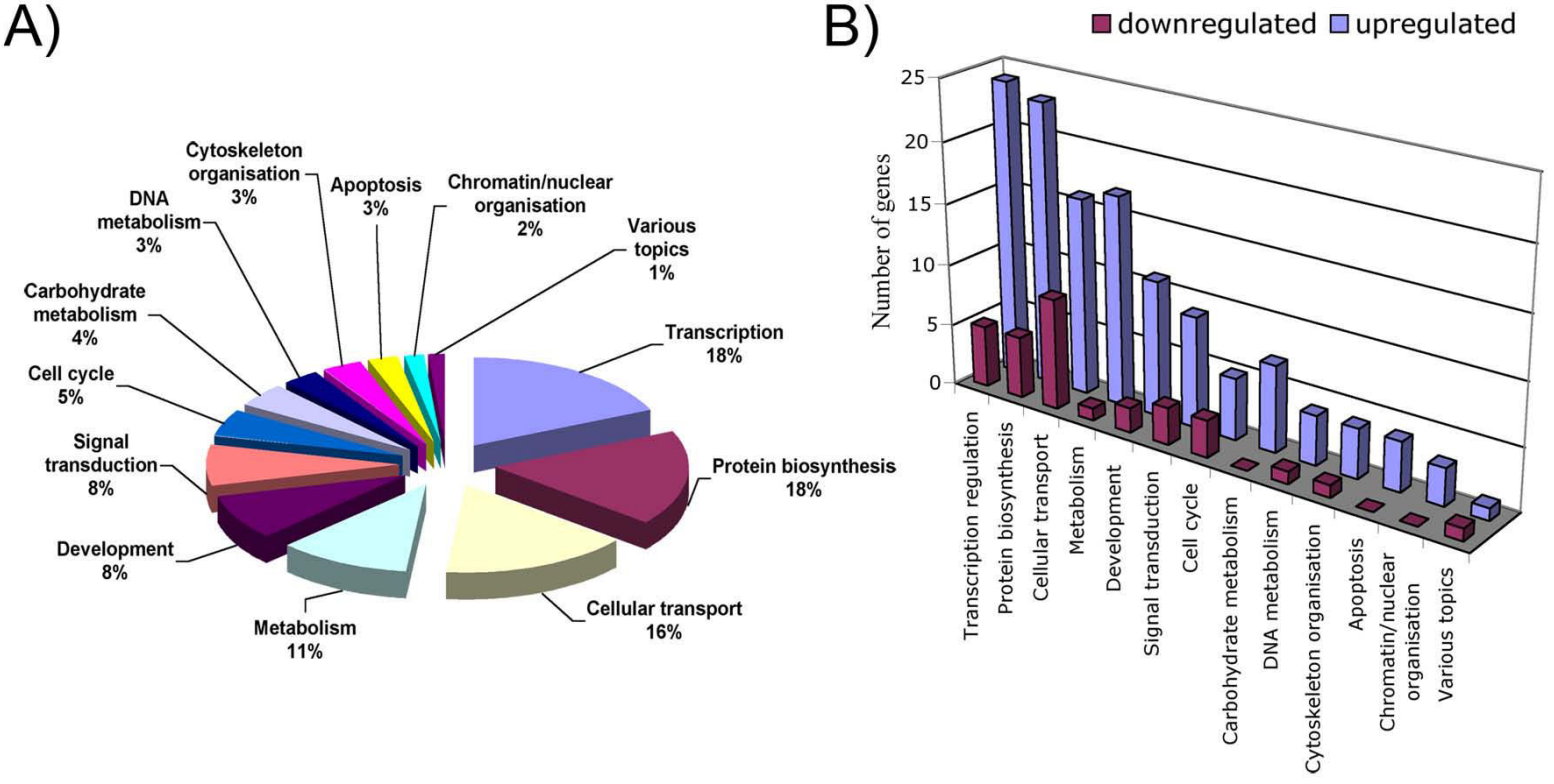
**Microarray-based analysis of the transcription profiles of MII^NSN ^and MII^SN ^oocytes**. (A) Twelve major biological processes that characterise the group of regulated and annotated genes. (B) The number of down-regulated and up-regulated genes in each of the twelve biological processes.

The microarray platform that we used allowed the analysis of a total of 39,450 genes and gene sequences (from now on they will be all referred to as genes). Of these, 1,421 genes were expressed only in MII^NSN ^oocytes; 1,967 genes were expressed specifically in MII^SN ^oocytes and 5,646 genes were expressed in both types of oocytes. A fold-change cut-off of at least 1.5 and a p value < 0.05 was set to filter the genes whose expression was differentially regulated. In the comparison between MII^NSN ^and MII^SN ^oocytes, we identified a total of 380 regulated genes, of these 303 were up-regulated in MII^NSN ^oocytes, and 77 down-regulated (Additional file 1, see the 'MII-NSN vs. MII-SN' data sheet). Using the Gene Ontology (GO) annotations implemented in the Illumina platform (Illumina^®^, 2007) and also by DAVID (Database for Annotation, Visualization and Integrated Discovery) [45], we identified 12 major biological themes that characterised the group of regulated and annotated genes (Figure 3A): transcription regulation (18%, mainly negative regulation), protein biosynthesis (18%), cellular transport (16%), metabolism (11%), development (8%), signal transduction (8%), cell cycle (5%, mainly negative regulation), carbohydrate metabolism (4%), DNA metabolism (3%), cytoskeleton organisation (3%), apoptosis (3%), chromatin/nuclear organisation (2%) and various GO (1%); also, some genes fell into a group with undetermined GO (162 unannotated genes). An inherent weakness of the bioinformatic programs available to analyse the patterns of gene expression obtained following microarrays analysis is that they are limited to those genes with assigned annotations or published relationships, and depend on the accuracy of these annotations. The number of down-regulated and up-regulated genes in each of the 12 categories and the list of these genes are shown in Figure 3B and Additional file 2, respectively.

To determine whether and how individual genes are inter-related or interact with each other and to search for biological pathways and the inter-relationships between network genes, we used the Ingenuity Pathways Analysis (IPA) version 5.5 analysis tool (Ingenuity^® ^Systems, ). The genes of each gene group (called focus genes) were uploaded as tab-delimited text files and IPA queried the Ingenuity Pathways Knowledge Base for interactions between focus genes and all other objects stored in the knowledge base and generated a set of networks. Those networks with a score ≥ 2 were considered for further analyses (see Additional File 3 for details).

#### Genes up-regulated in MII^NSN ^oocytes

The great majority (79.7%) of the regulated genes was up-regulated. When IPA was performed on the up-regulated focus genes, it generated 10 networks (Table 1) with a score of 2 or higher, of these, 9 had 10 or more focus genes (35 is the highest number of genes allowed in each network). Because a score of 2 or higher has a confidence of at least 99% of not being generated by chance, the high confidence level obtained, particularly in the top nine networks, reveals the strong interrelation and interaction among the genes that are up-regulated in MII^NSN ^oocytes. Figure in Additional file 4 shows the top 9 gene expression networks obtained. The specificity of these networks to the biology of the ovary was confirmed using IPA to enquire public ovarian gene expression data sets. Network 1 has 23 focus genes and of these 21 (91.3%) are known to be expressed in ovarian tissue. In networks 2–9, the percentage of ovarian genes decreased from 85.7% (network 4) to 50% (network 7). Comparison with data sets of somatic tissues, namely liver, kidney, heart, lung and spleen gave a lower matching (data not shown).

**Table 1:** Networks generated from IPA for focus genes that are up-regulated in MII^NSN ^oocytes when compared to MII^SN ^oocytes.*

**Network**	**Genes in ** **network**	**Score**	**Focus ** **genes**	**Top ** **functions**
1	*Akt, Anxa2, Bhlhb2, Calpain, Crkl, Glb1*, ***Hoxa7***, *Insulin, Integrin, Lasp1, Ldl, Mcl1, Mdh2, Mfn2, Nadh2 Dehydrogenase, Nadh2 Dehydrogenase (Ubiquinone), Ndufa1, Ndufa3, Ndufv1, Pdgf Bb, Pi3k*, ***Pik3c3***, *Pkc(S), Pp2a, Ptpn1, Rac, Rpl27a, Rps6*, ***Rps18***, *Slc3a2, Smg5, Strn3, Tmsb10, Tsc2, Tspan3*	42	23	Cancer, Immunological Disease, Tumor Morphology
2	*Bat3, Bcas3, Calmodulin, Crkrs, Egfr, Helz, Histone H3, Hsp70, Ing1, Invs*, ***Iqgap1****(Includes Eg:8826), Map3k12, Msse, Nbpf15, Nelf*, ***Nr2e1***, *P38 Mapk, Polr2l (Includes Eg:5441), Prkg1*, ***Ranbp1***, *Rap1, Rbm16, Rna Polymerase Ii, Setd2, Slc9a8, Smn1, Spag7, Spt1, Tarbp1*, ***Terf2ip***, *Tgf Beta, Ubc, Uqcrb, Uqcrc1, Wdr36*	26	16	Post-Translational Modification, Cancer, Genetic Disorder
3	*Abpa (Includes Eg:11354), Amino Acids, Arrb2, Cebpa, Clta, Cltb*, ***Ddx21***, *Eef1a2, Farp2, Fntb, Glipr1, Grasp, Grin3b, Grina, H1fx, Ifitm2, Insr, Mark3, Myo6, Nmda Receptor, Nusap1, Paip2, Pfkfb3, Pim1, Prkx, Sag, Snrk, Src, Stk38, Stk17a, Tfam, Tk2, Tp53, Tp53rk, Vegf*	22	14	Amino Acid Metabolism, Post-Translational Modification, Small Molecule Biochemistry
4	*Afg3l2, Arl2, Atf7ip, Atox1, Atp13a2, Atp13a5, Atp5a1, Atp6v0a1, Atp6v0b, Atp6v0e2, Atp6v1c2, Atp6v1d, Atp6v1e2, Atp6v1g2, Atp6v1h, Atpase, Ddx19b, Ddx3y (Includes Eg:26900), Dqx1, Eif3a, Eif3f, Eif3j, Eif4e, Gtp, H+-Transporting Two-Sector Atpase, Lsm10 (Includes Eg:84967), Mlh1, Psmc4, Psmc5*, ***Psmd7***, *Ralbp1, Rfc4, Rsf1, Spast, Tcirg1*	22	14	Molecular Transport, Protein Synthesis, Gene Expression
5	*Acaa2, Acaa1b, Acsl4, Alad, Bcat1, Cidec, Cpt2, Ddx11, Dleu1, Dleu2, Dnmt3l, E2f1, Emp1, Erbb2*, ***Exosc9***, *Fng, Glg1, Gtf3c2, Hdac1 (Includes Eg:3065)*, ***Hn1***, *Lxn, Mfap1, Mfng, Myc, Notch1, Psat1, Rfng, Rps16, Rps20, Snrpn, Sycp3, Tbp*, ***Tle4***, *Tmem126a, Uxt*	20	13	Cancer, Dermatological Diseases and Conditions, Cell Cycle
6	*Aars, Arsb, Arsd, Arse, Arsf, Arsg, Arsi, Arsj, Cct5, Cxcl12, Cxcr6 (Includes Eg:10663), Eif4h, Gns, Golga3, Hsd17b10, Kdelr2, Loxl2, Nsdhl, Pls3, Pth, Rab33b*, ***Rbm14***, *Rdh11, Sec61a1*, ***Sec61g***, *Slc34a1, Slc34a2, Srebf1, Ssr2, Stch, Sulf2*, ***Sumf1***, *Syvn1, Tgfb1, Xbp1*	20	13	Tissue Development, Tissue Morphology, Cellular Function and Maintenance
7	*Abtb1, Actin Alpha-Ca2+-Myosin-Tropomyosin-Troponinc-Troponin I-Troponin T, Adcy9, Beta-Estradiol, Ca2+, Clec3b, Cnn3, Fn1, Ifi30, Igf1, Kcnn2, Mek1/2, Myh2, Nmur2, Nudt1, Pcna, Pdlim2, Pfkl, Pkia, Pla2g12a, Pla2g2d, Plg, Poldip2, Psma2*, ***Psmb2***, *Psmd6, Psmd14, Pten, S100a2, Saps2, Serpinb12, Slc6a9, Spint1, Trpv2, Tsta3*	18	12	Cancer, Cellular Growth and Proliferation, Reproductive System Disease
8	*Alp, Aprt, Atp1a3, Atp5l, Bmp2, Chgb, Cox7a1, Creb1, Esd, Fgf7, Fos, Foxm1*, ***Gfra1***, *Hcg 25371, Hgs, Hr, Klf3, Me1, Myo5b, Nfe2l2, Nkx6-2, Nudt7, Pgd, Phyh, Psmc3ip, Punc, Rxrb, Smarcd2, Tcea3, Thrb, Thyroid Hormone, Trim3*, ***Trip4***, *Trip11, Ttf1*	16	11	Gene Expression, Connective Tissue Development and Function, Cellular Development
9	*Anapc5, Aptx, Cdca5, Cdca7l, Cdkn2a, Ctbp2, Ddx55, Deaf1, Epb42, Gypa, Icos, Ipo13, Ldb1, Lmo2, Lmo4, Mdm4, Nfkbib, Nhlh1, Nr3c1, P1, P4-Di(Adenosine-5') Tetraphosphate, Pdcd2, Pex3, Pex19, Plagl1, Rpl5, Sae1, Skp1, Smad2/3-Smad4, Sod2, Tcf3, Tfap2b, Tfap2c, Tgif1, Ube2i, Zp2*	14	10	Gene Expression, Cell Cycle, Skeletal and Muscular System Development and Function
10	*C4bpa, Foxj2*	2	1	Cell-To-Cell Signaling and Interaction, Hematological System Development and Function, Tissue Development

*: in bold are Oct-4-regulated genes (see below).

The gene expression networks described were associated to top functions such as gene expression, protein synthesis, cancer, immunological disease, genetic disorder, dermatological disease and reproductive system disease, highlighting the complexity of the different transcriptional profile between developmentally incompetent MII^NSN ^and competent MII^SN ^oocytes.

IPA was then interrogated to obtain a list of biochemical pathways that are representative of the functions in which the genes are involved. The genes up-regulated in MII^NSN ^oocytes were assigned to more than 80 pathways; 11 of these were considered statistically significant (see Additional file 3) and the top six, together with the gene expression networks they belong to, are shown in Table 2. These six pathways included a total of 15 different genes, the majority of which belonged to network 1 (4 genes), network 2 (4 genes) and network 4 (7 genes), and were associated with oxidative phosphorylation, mitochondrial dysfunction, galactose metabolism, purine metabolism, protein ubiquitination and GABA receptor signalling. Some of these genes are expressed within different pathways.

**Table 2:** Top six pathways generated from IPA for focus genes that are up-regulated in MII^NSN ^oocytes and assigned to their respective gene networks.

Pathway	Gene Symbol	Description	Network	Location	Type
Oxidative Phosphorylation	*Ndufa1*	NADH dehydrogenase (ubiquinone) 1 alpha subcomplex, 1, 7.5 kDa	1	Cytoplasm	enzyme
	*Ndufa3*	NADH dehydrogenase (ubiquinone) 1 alpha subcomplex, 3, 9 kDa	1	Cytoplasm	enzyme
	*Ndufv1*	NADH dehydrogenase (ubiquinone) flavoprotein 1, 51 kDa	1	Cytoplasm	enzyme
Mitochondrial Dysfunction	*Ndufa3*	NADH dehydrogenase (ubiquinone) 1 alpha subcomplex, 3, 9 kDa	1	Cytoplasm	enzyme
	*Ndufv1*	NADH dehydrogenase (ubiquinone) flavoprotein 1, 51 kDa	1	Cytoplasm	enzyme
Galactose Metabolism	*Glb1*	galactosidase, beta 1	1	Cytoplasm	enzyme
Oxidative Phosphorylation	*Uqcrb*	ubiquinol-cytochrome c reductase binding protein	2	Cytoplasm	enzyme
	*Uqcrc1*	ubiquinol-cytochrome c reductase core protein I	2	Cytoplasm	enzyme
Protein Ubiquitination	*Ubc*	ubiquitin C	2	Cytoplasm	other
GABA Receptor Signalling	*Ubc*	ubiquitin C	2	Cytoplasm	other
Mitochondrial Dysfunction	*Uqcrb*	ubiquinol-cytochrome c reductase binding protein	2	Cytoplasm	enzyme
	*Uqcrc1*	ubiquinol-cytochrome c reductase core protein I	2	Cytoplasm	enzyme
Purine Metabolism	*Polr2l*	polymerase (RNA) II (DNA directed) polypeptide L, 7.6 kDa	2	Nucleus	enzyme
Oxidative Phosphorylation	*Atp5a1*	ATP synthase, H+ transporting, mitochondrial F1 complex, alpha subunit 1, cardiac muscle	4	Cytoplasm	transporter
Oxidative Phosphorylation	*Atp6v0a1*	ATPase, H+ transporting, lysosomal V0 subunit a1	4	Cytoplasm	transporter
	*Atp6v0b*	ATPase, H+ transporting, lysosomal 21 kDa, V0 subunit b	4	Cytoplasm	transporter
Protein Ubiquitination	*Psmc4*	proteasome (prosome, macropain) 26S subunit, ATPase, 4	4	Nucleus	peptidase
	*Psmc5*	proteasome (prosome, macropain) 26S subunit, ATPase, 5	4	Nucleus	transcription regulator
	*Psmd7*	proteasome (prosome, macropain) 26S subunit, non-ATPase, 7	4	Cytoplasm	other
Purine Metabolism	*Atf7ip*	activating transcription factor 7 interacting protein	4	Nucleus	transcription regulator
	*Atp5a1*	ATP synthase, H+ transporting, mitochondrial F1 complex, alpha subunit 1, cardiac muscle	4	Cytoplasm	transporter
	*Atp6v0b*	ATPase, H+ transporting, lysosomal 21 kDa, V0 subunit b	4	Cytoplasm	transporter
	*Psmc4*	proteasome (prosome, macropain) 26S subunit, ATPase, 4	4	Nucleus	peptidase
	*Psmc5*	proteasome (prosome, macropain) 26S subunit, ATPase, 5	4	Nucleus	transcription regulator

#### Genes down-regulated in MII^NSN ^oocytes

IPA analysis of the focus genes down-regulated in MII^NSN ^oocytes generated 7 networks (Additional file 5 and Additional file 6) with a score ≥ 2 (the first three networks with a score of 10 or higher are represented in Additional file 7). The top functions associated with these gene expression networks include cell cycle, cell death, embryonic development and cancer. When IPA was interrogated to generate the biochemical pathways that were most representative of the functions attributed to the genes involved in the 7 networks, it created ten major pathways represented by 7 genes, 5 of which are associated with network 1 (2 genes) and network 2 (3 genes) (Additional file 8). Of these 5 genes, *Atm *and *Mdm2 *(a kinase and a transcription regulator, respectively) are involved in the G_2_/M DNA damage checkpoint regulation. The protein encoded by the *Prdx6 *gene is a member of the thiol-specific antioxidant protein family, involved in the regulation of phospholipids turnover as well as in the protection against oxidative injury.

To identify the inter-relationship among up- and down-regulated genes, we next carried out an IPA analysis of all the regulated genes.

#### Genes regulated in MII^NSN ^oocytes

The IPA of all the regulated genes, documented 25 gene expression networks with a score ≥ 2, of these 12 showed a score ≥ 12 (Additional file 9 and Additional file 10). Since most of the regulated genes are up-regulated (79.7%), as expected, the top functions represented by these networks are those already identified with the separated analysis of the up- and down-regulated genes (e.g., cell cycle, cellular compromise, cell death, cancer, cellular growth and proliferation found in networks 1–3), but it also revealed new categories (e.g., post-translational modification, connective tissue development, found in networks 2 and 3). In network 1, NF-kB is one of the principal nodes, together with insulin, calmodulin and protein phosphatase 2A (PP2A). Correct expression of NF-kB is required for the development of the mouse preimplantation embryo, as inhibition of its expression blocks development at the 2-cell stage [46]. PPA2 is one of the four major Ser/Thr phosphatases implicated in the negative control of cell growth and division.

Further analysis of the most representative biochemical pathways identified nine top pathways, 6 of which are shown in Additional file 11. The most representative pathways were those assigned to protein ubiquitination, oxidative phosphorylation, ubiquinone biosynthesis, G_2_/M DNA damage checkpoint regulation, fructose and mannose metabolism, valine, leucine and isoleucine degradation, attributed mainly to network 1 (8 genes), network 3 (6 genes) and network 6 (5 genes).

#### Genes expressed solely in MII^NSN ^or MII^SN ^oocytes

The comparison of the expression profiles of MII^NSN ^with that of MII^SN ^oocytes, resulted in the identification of a small number of focus genes transcribed exclusively by MII^NSN ^oocytes (29 genes, 8 annotated) (Additional file 1, see the 'Only in MII-NSN' data sheet) or MII^SN ^oocytes (7 genes, 3 annotated) (Additional file 1, see the 'Only in MII-SN' data sheet). Three of the MII^NSN ^oocyte-specific genes have an assigned GO function as negative regulators of cell proliferation (*Ogfr *and *Mll1*) or apoptosis (*Spata4*), whereas two annotated MII^SN ^oocyte-specific genes are involved in processes of positive regulation of the cell cycle (*Crebbp *and *Fank1*). Out of 29 focus genes expressed only in MII^NSN ^oocytes, 10 were assigned to a single gene expression network (Additional file 12, genes in bold; Additional file 13), with top functions that included hair and skin development and function, organ morphology and cellular development. The number of MII^SN^-specific genes was so low that it was not considered for further analysis.

RT-PCR analysis of the relative number of transcripts of two MII^NSN ^oocyte-specific genes (*Jam2 *and *Ogfr*) and two MII^SN ^oocyte-specific genes (*Crebbp *and *Fnip1*) confirmed the microarrays data and the presence of a different number of transcripts in the two types of oocytes; however, whilst *Jam2 *and *Ogfr *were shown to be expressed only in MII^NSN ^oocytes (Additional file 14); *Crebbp *and *Fnip1 *were not exclusive to MII^SN ^oocytes, but instead showed respectively a 1.4-fold and 1.9-fold, higher number of transcripts in these gametes (Additional file 14). The presence of transcripts of *Crebbp *and *Fnip1 *genes in MII^NSN ^oocytes is due to the overt single-cell sensitive of nested-PCR that amplified the low number of transcripts that were not detected by the less sensitive microarray analysis. When the number of the second PCR cycles was reduced to 22 (*Crebbp*) and 20 (*Fnip1*), expression was not detected in the MII^NSN ^oocyte samples.

In summary, the results of the microarray analysis as a whole, indicate that developmentally incompetent MII^NSN ^oocytes have activated, among others, gene expression networks implicated in the regulation of biochemical pathways representative of the adverse biological status of these oocytes. These pathways include, oxidative phosphorylation, mitochondrial dysfunction and apoptosis.

To identify Oct-4 target genes among the list of regulated genes, we compared our list with the published mouse and human Chip datasets of Oct-4-regulated genes [32,43].

### Eighteen Oct-4-regulated genes are associated with adverse gene expression networks in developmentally incompetent MII^NSN ^oocytes

**Figure 4:**
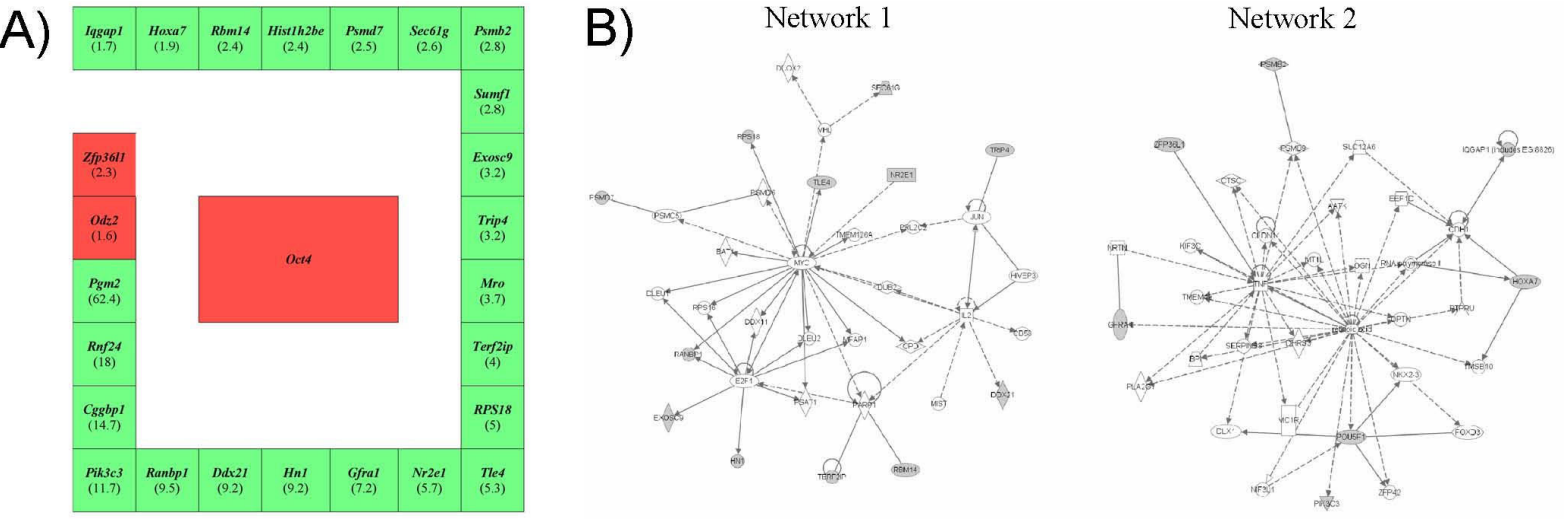
**Down-regulation of Oct-4 expression in MII^NSN ^oocytes induces the up-regulation of a group of Oct-4-regulated genes associated with gene expression networks involved in the activation of adverse pathways**. (A) Twenty-five Oct-4-regulated genes present in the list of regulated genes determined with our microarray analysis. Twenty-three of them are up-regulated (green square), two are down-regulated (red square). Within each square the name of the gene and the n-fold up- or down-regulation are reported. (B) The two top gene expression networks generated when IPA was interrogated with the list of these 25 Oct-4-regulated genes. Grey symbols are focus genes (use the zoom in tool to enlarge the networks).

This analysis identified a total of 25 target genes. When comparing MII^NSN ^with MII^SN ^oocytes, all these genes, with the exception of *Odz2 *and *Zfp36l1 *(down-regulated 1.6 and 2.3-fold, respectively), were up-regulated in MII^NSN ^oocytes with fold changes ranging from 1.7 (*Iqgap1*) to 62.4 (*Pgm2*) (Figure 4A). Eighteen of these genes were part of gene expression networks 1 to 8 generated from the IPA of up-regulated genes in MII^NSN ^oocytes (Table 1, genes highlighted in green), and thus involved in the activation of the same biochemical pathways described above. When IPA was specifically interrogated with the list of these 25 genes, including Oct-4, it generated 6 networks (Table 3) with a score ≥ 2, of these, the first two contained 12 and 7 (including *Oct-4 *itself) focus genes respectively, and had top functions centred around biological themes such as gene expression, cell death, cancer and reproductive system disease. *Oct-4 *was part of network 2 (Figure 4B), which included the TNF (tumor necrosis factor) pathway, a multifunctional proinflammatory cytokine that acts on several different signalling pathways to regulate apoptosis (directly activating the caspase cascade or through a mitochondria-mediated apoptosis [for a review see [47]]), NF-kB activation of inflammation, and activation of stress-activated protein kinases (SAPKs).

**Table 3:** Networks generated from IPA for Oct-4-regulated genes.*

**Network**	**Molecules ** **in Network**	**Score**	**Focus ** **Molecules**	**Top ** **Functions**
1	*Bat1, Cd58, Cpd, Ddx11*, ***Ddx21***, *Dleu1, Dleu2, Dub2, Duox2, E2f1, Exosc9, Hivep3*, ***Hn1***, *Il2, Jun, Mfap1, Mist, Myc*, ***Nr2e1***, *Parp1, Prl2c2, Psat1, Psmc5, Psmd6*, ***Psmd7***, ***Ranbp1***, ***Rbm14***, *Rps16*, ***Rps18***, ***Sec61g***, ***Terf2ip***, ***Tle4***, *Tmem126a*, ***Trip4***, *Vhl*	28	12	Gene Expression, Cell Death, Hematological Disease
2	*Aatk, Bpi, Cdh1, Cldn11, Ctsc, Dhrs3, Dlx1, Eef1d, Foxd3*, ***Gfra1***, ***Hoxa7***, ***Iqgap1****(Includes Eg:8826), Kif3c, Mc1r, Mt1l, Nif3l1, Nkx2-3, Nrtn, Ogn, Optn*, ***Pik3c3***, *Pla2g7, Pou5f1*, ***Psmb2***, *Psmb9, Ptpru, Retinoic Acid, Rna Polymerase Ii, Serpinb8, Slc12a6, Tmem49, Tmsb10, Tnf, Zfp42*, ***Zfp36l1***	15	7	Cancer, Reproductive System Disease, Cell Death
3	*Cd79a*, ***Mro***	3	1	Lipid Metabolism, Small Molecule Biochemistry, Viral Function
4	***Cggbp1***, *Fmr1*	3	1	Connective Tissue Development and Function, Developmental Disorder, Genetic Disorder
5	*Pgm*, ***Pgm2***, *Pmm*	3	1	Carbohydrate Metabolism, Small Molecule Biochemistry
6	*Arsa, Arsb, Arsd, Arse, Arsf, Arsg, Arsi, Arsj, Gns, Ids, Sgsh, Sts, Sulf1, Sulf2*, ***Sumf1***	2	1	Genetic Disorder, Metabolic Disease, Skeletal and Muscular System Development and Function

*: In bold are the Oct-4-regulated focus genes. Underlined is the *Oct-4 *(*Pou5f1*) gene.

Interestingly, *Zfp36l1 *(Zinc finger protein 36), one of the two Oct-4-regulated genes that was down-regulated in MII^NSN ^oocytes, is a member of the *Tis11 *family of early response genes, of which *Zfp36l2*, another maternal-effect gene crucial for early embryonic development, is also a member [48]. Perhaps, *Zfp36l1 *is a novel maternal-effect gene whose expression is regulated by Oct-4 and whose down-regulation may affect the developmental competence of MII^NSN ^oocytes.

The microarray analysis revealed a 1.8-fold down-regulation of *Oct-4 *expression in MII^NSN ^oocytes. However, this data was not included in the list of regulated genes (Additional file 1) because its *p *value was equal to 0.051, thus just slightly above the set cut-off limit of p < 0.05. To understand how the expression of the *Oct-4 *gene was related with the adverse gene expression networks that we found activated in MII^NSN ^oocytes, we next included the expression data for *Oct-4 *obtained by microarray analysis (despite its *p *value was 0.051) into the list of up-regulated or down-regulated genes and performed an IPA analysis. With the group of down-regulated genes, *Oct-4 *was included in network 1 (which has top functions as gene expression, cancer and cell cycle; see Additional file 5); with the up-regulated genes, *Oct-4 *was integrated into network 2, which has top functions as post-translational modification, cancer, genetic disorder and activates adverse pathways such as oxidative phosphorylation and mitochondrial dysfunction, see Table 1 and Table 2). In both cases the gene fell into the two top networks highlighting the central role it plays.

In summary, down-regulation of Oct-4 expression in MII^NSN ^oocytes induces the up-regulation of a group of 18 Oct-4-regulated genes that are part of the top gene expression networks involved in the activation of adverse pathways, i.e., oxidative phosphorylation, mitochondrial dysfunction and apoptosis.

## Discussion

The maternal contribution of transcripts and proteins supplied to the zygote is crucial for the successful progression of preimplantation development. An aberrant oocyte inheritance leads to developmental arrest, mostly at the time of ZGA. To gain a better understanding of the genes, gene expression networks and biochemical pathways that may be activated/inactivated to compromise the developmental competence of MII oocytes, we compared two types of mouse MII oocytes, one which is developmentally competent and reaches full term development (MII^SN ^oocyte), the other that ceases development at the 2-cell stage (MII^NSN ^oocyte).

Our study began with the analysis of the profile of expression of five maternal-effect genes. We showed that of these, only the expression of *Stella *was mis-regulated and its protein absent in MII^NSN ^oocytes. The maternal inheritance of Stella is needed for correct preimplantation development [22]. When oocytes from *Stella*^-/- ^females are inseminated with wild-type sperm, while fertilisation proceeds normally, development is severely altered resulting in a progressive decline in the number of blastocysts, with the majority of embryos that do not develop beyond the 2-cell stage. *Stella *encodes for a basic protein with a SAP-like domain and a splicing factor-like motif thought to have a role in chromosomal organisation or RNA metabolism. Stella binds both RNA and DNA *in vitro*, supporting its involvement in linking chromatin with RNA-related processes, as it is the case for other SAP-domain proteins [49]. A recent study has shown that Stella protects against inappropriate DNA demethylation in the maternal genome and at certain imprinted loci during epigenetic reprogramming after fertilisation [50]. Down-regulation of Stella, is likely to be partly causal of the developmental arrest at the 2-cell stage of MII^NSN ^oocytes. Lack of Stella protein in MII^NSN ^oocytes is a consequence of the down-regulation of Oct-4, which is known to regulate *Stella *expression in ES and EC cells. This implies a pivotal role for maternal Oct-4 in the establishment of the developmental legacy of female gametes. The down-regulation of Oct-4/Stella in NSN GV oocytes compromises their developmental competence.

In favour of the hypothesis- "factors present in MII^SN ^oocytes, but absent in MII^NSN ^oocytes, govern their developmental competence", Inoue and collaborators [18] have micromanipulated fully grown SN and NSN antral oocytes to demonstrate that the developmental competence of SN oocytes is dependent on unknown cytoplasmic factor(s) that are released from the germinal vesicle at the time of MII formation and that these ooplasmic factors are not contained in the GV of NSN oocytes. Both Stella and Oct-4 proteins localise within the germinal vesicle of the SN antral oocytes; following meiosis resumption and germinal vesicle break-down, Oct-4 is circumscribed mainly around the MII-plate area, while Stella is released within the ooplasm. The association of Oct-4 (and Stella) with the nucleolus in antral SN oocytes may have a functional significance. It was previously shown that Oct-4 is closely associated with RNA polymerase II in the nucleolus of oocytes (nucleolus-like bodies, NLBs) [51]; since NLBs are the only coilin-containing structures at the end of pre-ovulatory stages of oogenesis, they may represent sites for the storage of the transcriptional machinery needed after fertilisation and of which Oct-4 may be a key component [51]. This association of Oct-4 with the NLBs may secure a transfer of the maternally inherited protein through the NLB to the zygote [51,52], where Oct-4 perhaps continues to be necessary as a regulator of the molecular events leading to ZGA.

Our microarray analysis indicate that the role of Oct-4 as a regulator of gene expression in MII oocytes extends beyond the regulation of *Stella *expression and has an inhibitory effect on the expression of Oct-4-regulated genes in developmentally competent MII^SN ^oocytes. Down-regulation of Oct-4 in MII^NSN ^oocytes correlates with the up-regulation of 23 Oct-4-regulated genes involved in the activation of gene expression networks and biochemical pathways that most represent the negative legacy of MII^NSN ^oocytes. These pathways include oxidative phosphorylation, mitochondrial dysfunction and cell death. Following fertilisation, these negative features might be passed on to the zygote and constitute a detrimental maternal legacy that affects further development.

Oxidative phosphorylation and mitochondrial dysfunction are biochemical pathways principal to the first two top gene expression networks described for the group of up-regulated genes (these represent the majority of the regulated genes, 79.7%), which emphasises the central role that these genes and mitochondria play in the biochemistry of the developmentally incompetent MII^NSN ^oocytes. In addition to producing most of the cell's ATP, recent studies have demonstrated that mitochondria have a central role in cellular processes such as the regulation of intracellular redox potential and control of apoptosis in oocytes and preimplantation embryos [for a review see [53]]. Accumulation of reactive oxygen species (ROS) induces apoptosis in mouse zygotes, and mitochondria are involved in the early phase of oxidative stress-induced apoptosis [54], that leads to developmental arrest and alterations of the ZGA [55,56]. Also, changes in the levels of ROS regulate a number of transcription factors critical in early development, these include PEBP2, AP-1, p53 and NF-kB [57-59]. The two-fold role for mitochondria, to maintain life or to commit to cell death, may represent a quality control system in the female gamete and in the early embryo that will determine whether the embryo proceeds further into development or is quickly eliminated [53].

If down-regulation of *Oct-4 *activates these negative pathways, what does upstream of *Oct-4 *regulate its expression? We have previously shown an effect of PMSG (a gonadotropin which mimics an FSH action) on *Oct-4 *gene expression during folliculogenesis, demonstrating a hormonal regulation of oocyte-specific gene expression [39]. PMSG, which was used in this study to prime females before oocytes isolation, may enter the gamete with the involvement of a FSH-R signaling pathway that, through the modulation of c-kit on the surface of oocytes [60], conveys the gonadotropin signal into the nucleus and then act upon the transcription of several genes. One candidate could be GCNF (germ cell nuclear factor), a member of the nuclear receptor family, critical in controlling basal activities or hormonal responsiveness of numerous target genes and known to repress *Oct-4 *expression [37]. GCNF may be a target of PMSG action that leads to the down-regulation of *Oct-4 *expression in MII^NSN ^oocytes. This hypothesis needs to be tested, however, it is interesting that in our microarray analysis, *GCNF *was 1.6-fold up-regulated in MII^NSN^, although, because of its *p *value > 0.05, it was excluded from the list of regulated genes.

## Conclusion

In summary, our study of developmentally competent and incompetent mouse MII oocytes has identified a small number of genes, gene expression networks and biochemical pathways whose activation is critical for the correct progression of the early stages of preimplantation development and regulate the 2-cell block in MII^NSN ^oocytes. Our data constitute a framework for further exploration of the genes and related transcriptional networks dedicated to establishing and preservating the developmental competence of mammalian oocytes. Based on our results, we have drawn a working hypothesis that assigns a role to some of the molecular players that have been described in this and other studies. The use of a model study in which the MII oocyte ceases development consistently at the 2-cell stage has allowed to illustrate a role for the maternal *Oct-4 *that has never been described before. Oct-4 emerges as a key regulator of the molecular processes that govern the establishment of the maternal legacy of MII oocytes required for the transition from a gametic to an embryonic control of development. A sound knowledge of the orchestrating molecules and the biochemical pathways entailed during the very early stages of development will allow a better understanding of the molecular mechanism(s) that determine the block of development at the time of ZGA, which in humans is the cause of a loss of a high percentage of embryos obtained by *in vitro *fertilisation. A thorough functional analysis of these genes in preimplantation embryos derived from MII^NSN ^and MII^SN ^oocytes is our next challenge.

## Methods

### Oocytes isolation and maturation

Four to six week-old B6C3F1 female mice (Charles River, Como, Italy) were injected with 3.5 I.U. PMSG (Pregnant Mare Serum Gonadotropin, Folligon, Intervet Srl, Italy) and sacrificed 48 hr later. Ovaries were isolated and antral oocytes with a diameter > 70 nm were collected in M2 medium by puncturing the ovarian surface with a sterile needle, they were transferred into subsequent washes of M2 medium, until they appeared, under an inverted microscope, completely free of cumulus cells. Then, they were classified into NSN and SN oocytes and matured to the MII stage (for details, see Additional file 3 and [61]). The majority of SN (85%) and NSN (65%) antral oocytes matured to the MII stage. Approximately 50 MII^NSN ^or MII^SN ^oocytes were transferred in single 0.2 ml eppendorf tubes containing 50 μl Trizol Reagent (Invitrogen), for microarray analysis. For RT-PCR (Retro Transcription-Polymerase Chain Reaction) analysis, individual MII oocytes were transferred in 0.5 μl medium to 0.2 ml eppendorf tubes containing 1.5 μl lysis buffer [17].

### RNA isolation and RT-PCR amplification

Relative amounts of transcripts of the five maternal-effect genes (*Zar1*, *Stella*, *Smarca4*, *Npm2*, and *Prei3*) and of the MII^NSN^-specific (*Jam2 *and *Ogfr*) and MII^SN^-specific (*Crebbp *and *Fnip1*) genes were determined by using a semi-quantitative RT-PCR assay (for a detailed description of the assay, see Additional file 3 and ref. 39). For each experiment, at least 5 single MII^NSN ^or MII^SN ^oocytes were analysed. Statistical analysis was done using the SigmaStat 3.0 software and comparing samples with the *t*-test. Differences were considered significant when *p *< 0.05.

### Immunocytochemistry

Oocytes were fixed with freshly prepared 4% paraformaldehyde for 30 min and then permeabilised with 0.5% Triton X-100 for 15 min at 4°C. To suppress non-specific binding of antibodies, the gametes were incubated with 0.5% blocking reagent (Roche, Boston, MA) in TNT buffer (0.1 M Tris-HCl, pH 7.5, 0.15 M NaCl, 0.05% Tween-20) for 20 min at 4°C. Immunostaining was performed with rabbit anti-Oct-4 polyclonal antibody (Stanta Cruz Biotechnology; sc 9081, 1:500 dilution) or mouse anti-Stella monoclonal antibody (Millipore; MAB 4388, 1:1000 dilution). Oocytes were incubated with primary antibodies for 1 h at 37°C. The primary antibodies were detected using appropriated secondary antibodies diluted in TNT for 1 h at 37°C: Alexa Fluor488-goat anti-rabbit IgG (Molecular Probes; 1:6000 dilution) for Oct-4 detection and Alexa Fluor488-goat anti-mouse IgG (1:10000 dilution) for Stella detection. After immunostaining, oocytes were washed in three changes of TNT for 15 min at 4°C, and then counterstained with DAPI (0.2 μg/ml in PBS for 5 min) and mounted in Vectashield (Vector).

### Illumina bead chip hybridisations

Two independent pools of MII^NSN ^or MII^NSN ^oocytes consisting of approximately 50 oocytes per pool were used as the basis of our study. Messenger RNA was isolated using Oligo-dT-linked magnetic beads as described in Adjaye et al. [62,63]. In order to generate enough RNA for the subsequent microarray analysis, a two-round linear amplification protocol was adopted to generate biotin-labelled cRNA employing a linear amplification kit (Ambion, Austin, TX, United States). 1.5 μg of cRNA was used for the hybridisation reaction. Washing, Cy3-streptavidin staining, and scanning were performed on the Illumina BeadStation 500 (Illumina, San Diego, CA, United States) platform using reagents and following protocols supplied by the manufacturer. cRNA samples were hybridised onto Illumina mouse-6 BeadChips.

### Bioinformatic analysis

All basic expression data analysis was carried out using the manufacturer's software BeadStudio 1.0. Raw data were background-subtracted and normalised using the "rank invariant" algorithm. Normalised data were then filtered for significant expression on the basis of negative control beads. Selection for differentially expressed genes was performed on the basis of arbitrary thresholds for fold changes plus statistical significance according to the Illumina t-test error model.

Gene annotation was first performed with the BeadStudio software, combined with DAVID annotation tool [45]. File management, automated annotation and other statistical analyses not reported in the paper were performed with Matlab^® ^Bioinformatics Toolbox. The networks and pathways were generated with Ingenuity Pathways Analysis (Ingenuity^® ^Systems, ; for details see Additional file 3).

## Authors' contributions

ZC conceived the study, participated in its design and coordination, participated to the bioinformatic analyses and worked to the drafting of the manuscript; VM did the immunocytochemistry analyses; LS, RB and MS did the bioinformatic analyses; MB did the RT-PCR analyses; TCB did the microarrays analysis; CAR worked to the elaboration of the study; SG conceived the study, participated in its design and coordination and worked to the drafting of the manuscript; JA conceived the study, participated in its design and coordination, did the microarrays analyses and worked to the drafting of the manuscript.

## Supplementary Material

Additional file 1 List of regulated genes in the comparison between MII^NSN ^vs. MII^SN^. Click here for file

Additional file 2 Based on their gene ontology, genes that were up-regulated or down-regulated in MII^NSN ^oocytes were assigned to twelve biological categories most representative of their function. Click here for file

Additional file 3 Additional Methods. Click here for file

Additional file 4 Top nine gene expression networks generated by IPA with the list of genes up-regulated in MII^NSN ^oocytes. Grey symbols are focus genes (use the zoom in tool to enlarge the networks). See Additional file 6 for the symbols legend. Click here for file

Additional file 5 Networks generated by IPA for focus genes that are down-regulated in MII^NSN ^oocytes when compared to MII^SN ^oocytes. Click here for file

Additional file 6 Legend of symbols used in the figures shown in Additional files 4, 7, 10 and 13. Click here for file

Additional file 7 Top three gene expression networks generated by IPA with the list of genes down-regulated in MII^NSN ^oocytes. Grey symbols are focus genes (use the zoom in tool to enlarge the networks). Click here for file

Additional file 8 Pathways generated by IPA for focus genes that are down-regulated in MII^NSN ^oocytes. Click here for file

Additional file 9 Networks generated by IPA for all the focus genes that are regulated in MII^NSN ^oocytes when compared to MII^SN ^oocytes. Click here for file

Additional file 10 Top twelve gene expression networks generated by IPA with the list of genes regulated in MII^NSN ^oocytes. Green symbols, genes up-regulated; red symbols, genes down-regulated (the intensity of the colour indicates the level of regulation) (use the zoom in tool to enlarge the networks). Click here for file

Additional file 11 Pathways generated by IPA for focus genes that are regulated in MII^NSN ^oocytes. Click here for file

Additional file 12 Networks generated by IPA for focus genes that are expressed exclusively MII^NSN ^and not in MII^SN ^oocytes. Click here for file

Additional file 13 Gene expression network 1 generated by IPA with the list of genes expressed solely in MII^NSN ^oocytes. Grey symbols are focus genes (use the zoom in tool to enlarge the networks). Click here for file

Additional file 14 The gel electrophoresis shows the product of amplification of two genes expressed solely in MII^NSN ^oocytes (*Ogfr *and *Jam2*) and two genes expressed more abundantly in MII^SN ^oocytes (*Crebbp *and *Fnip1*). *Gapdh*, endogenous control whose transcripts are present equally in the two types of oocytes; 1–3, three different single MII^NN ^oocytes; 4–6, three different single MII^NSN ^oocytes. ∅1, RT blank; ∅2, first PCR blank; ∅3, second PCR blank; M, low mass ladder marker. Click here for file

## References

[B1] Schultz RM (2002). The molecular foundations of the maternal to zygotic transition in the preimplantation embryo. Hum Reprod Update.

[B2] Minami N, Suzuki T, Tsukamoto S (2007). Zygotic gene activation and maternal factors in mammals. J Reprod Dev.

[B3] Zuccotti M, Garagna S, Merico V, Monti M, Redi CA (2005). Chromatin organisation and nuclear architecture in growing mouse oocytes. Mol Cell Endocrinol.

[B4] Mandl AM (1962). Preovulatory changes in the oocyte of the adult rat. Proc R Soc Lond (Biol).

[B5] Lefevre B, Gougeon A, Nome F, Testart J (1989). In vivo changes in oocyte germinal vesicle related to follicular quality and size at midfollicular phase during stimulated cycles in the cynomolgus monkey. Reprod Nutr Dev.

[B6] Crozet N, Motlik J, Szollosi D (1981). Nucleolar fine structure and RNA synthesis in porcine oocytes during early stages of antrum formation. Biol Cell.

[B7] Mattson BA, Albertini DF (1990). Oogenesis: Chromatin and microtubule dynamics during meiotic prophase. Mol Reprod Dev.

[B8] Parfenov V, Potchukalina G, Dudina L, Kostyuchek D, Gruzova M (1989). Oocyte nucleus and the karyosphere formation (electron microscopic and autoradiographic data). Gamete Res.

[B9] Debey P, Szollosi MS, Szollosi D, Vautier D, Girousse A, Besombes D (1993). Competent mouse oocytes isolated from antral follicles exhibit different chromatin organization and follow different maturation dynamics. Mol Reprod Dev.

[B10] Longo F, Garagna S, Merico V, Orlandini G, Gatti R, Scandroglio R, Redi CA, Zuccotti M (2003). Nuclear localization of NORs and centromeres in mouse oocytes during folliculogenesis. Mol Reprod Dev.

[B11] Wickramasinghe D, Ebert KM, Albertini DF (1991). Meiotic competence acquisition is associated with the appearance of M-phase characteristics in growing mouse oocytes. Dev Biol.

[B12] Can A, Semiz O, Cinar O (2003). Centrosome and microtubule dynamics during early stages of meiosis in mouse oocytes. Mol Hum Reprod.

[B13] Christians E, Boiani M, Garagna S, Dessy C, Redi CA, Renard JP, Zuccotti M (1999). Gene expression and chromatin organisation during mouse oocyte growth. Dev Biol.

[B14] Bouniol-Baly C, Hamraoui L, Guibert J, Beaujean N, Szollosi MS, Debey P (1999). Differential transcriptional activity associated to chromatin configuration in fully grown germinal vesicle mouse oocytes. Biol Reprod.

[B15] Miyara F, Migne C, Dumont-Hassan M, Le Meur A, Cohen-Bacrie P, Aubriot FX, Glissant A, Nathan C, Douard S, Stanovici A, Debey P (2003). Chromatin configuration and transcriptional control in human and mouse oocytes. Mol Reprod Dev.

[B16] Zuccotti M, Giorgi Rossi P, Martinez A, Garagna S, Forabosco A, Redi CA (1998). Meiotic and developmental competence of mouse antral oocytes. Biol Reprod.

[B17] Zuccotti M, Ponce RH, Boiani M, Guizzardi S, Govoni P, Scandroglio R, Garagna S, Redi CA (2002). The analysis of chromatin organisation allows selection of mouse antral oocytes competent for development to blastocyst. Zygote.

[B18] Inoue A, Nakajima R, Nagata M, Aoki F (2008). Contribution of the oocyte nucleus and cytoplasm to the determination of meiotic and developmental competence in mice. Hum Reprod.

[B19] Morisato D, Anderson KV (1995). Signaling pathways that establish the dorsal-ventral pattern of the Drosophila embryo. Annu Rev Genet.

[B20] Newport J, Kirschner M (1982). A major developmental transition in early Xenopus embryos: I. characterization and timing of cellular changes at the midblastula stage. Cell.

[B21] Tong ZB, Gold L, Pfeifer KE, Dorward H, Lee E, Bondy CA, Dean J, Nelson LM (2000). Mater, a maternal effect gene required for early embryonic development in mice. Nat Genet.

[B22] Payer B, Saitou M, Barton SC, Thresher R, Dixon JP, Zahn D, Colledge WH, Carlton MB, Nakano T, Surani MA (2003). Stella is a maternal effect gene required for normal early development in mice. Curr Biol.

[B23] Bortvin A, Goodheart M, Liao M, Page DC (2004). Dppa3/Pgc7/stella is a maternal factor and is not required for germ cell specification in mice. BMC Dev Biol.

[B24] Christians E, Davis AA, Thomas SD, Benjamin IJ (2000). Maternal effect of Hsf1 on reproductive success. Nature.

[B25] Gurtu VE, Verma S, Grossmann AH, Liskay RM, Skarnes WC, Baker SM (2002). Maternal effect for DNA mismatch repair in the mouse. Genetics.

[B26] Wu X, Viveiros MM, Eppig JJ, Bai Y, Fitzpatrick SL, Matzuk MM (2003). Zygote arrest 1 (Zar1) is a novel maternal-effect gene critical for the oocyte-to-embryo transition. Nat Genet.

[B27] Burns KH, Viveiros MM, Ren Y, Wang P, DeMayo FJ, Frail DE, Eppig JJ, Matzuk MM (2003). Roles of NPM2 in chromatin and nucleolar organization in oocytes and embryos. Science.

[B28] De Vries WN, Evsikov AV, Haac BE, Fancher KS, Holbrook AE, Kemler R, Solter D, Knowles BB (2004). Maternal beta-catenin and E-cadherin in mouse development. Development.

[B29] Bultman SJ, Gebuhr TC, Pan H, Svoboda P, Schultz RM, Magnuson T (2006). Maternal BRG1 regulates zygotic genome activation in the mouse. Genes Dev.

[B30] Bourc'his D, Xu GL, Lin CS, Bollman B, Bestor TH (2001). Dnmt3L and the establishment of maternal genomic imprints. Science.

[B31] Leader B, Lim H, Carabatsos MJ, Harrington A, Ecsedy J, Pellman D, Maas R, Leder P (2002). Formin-2, polyploidy, hypofertility and positioning of the meiotic spindle in mouse oocytes. Nat Cell Biol.

[B32] Loh YH, Wu Q, Chew JL, Vega VB, Zhang W, Chen X, Bourque G, George J, Leong B, Liu J, Wong KY, Sung KW, Lee CW, Zhao XD, Chiu KP, Lipovich L, Kuznetsov VA, Robson P, Stanton LW, Wei CL, Ruan Y, Lim B, Ng HH (2006). The Oct4 and Nanog transcription network regulates pluripotency in mouse embryonic stem cells. Nat Genet.

[B33] Babaie Y, Herwig R, Greber B, Brink TC, Wruck W, Groth D, Lehrach H, Burdon T, Adjaye J (2007). Analysis of Oct4-dependent transcriptional networks regulating self-renewal and pluripotency in human embryonic stem cells. Stem Cells.

[B34] Greber B, Lehrach H, Adjaye J (2007). Silencing of core transcription factors in human EC cells highlights the importance of autocrine FGF signaling for self-renewal. BMC Dev Biol.

[B35] Levasseur DN, Wang J, Dorschner MO, Stamatoyannopoulos JA, Orkin SH (2008). Oct4 dependence of chromatin structure within the extended Nanog locus in ES cells. Genes Dev.

[B36] Campbell PA, Perez-Iratxeta C, Andrade-Navarro MA, Rudnicki MA (2007). Oct4 targets regulatory nodes to modulate stem cell function. PLoS ONE.

[B37] Cavalieri F, Scholer H, Lanza R, Gearhart J, Hogan B, Melton D, Pedersen R, Thompson J, West M (2005). Molecular facets of pluripotency. Stem cells.

[B38] Pesce M, Schöler HR (2000). Oct-4, control of totipotency and germline determination. Mol Reprod Dev.

[B39] Monti M, Garagna S, Redi CA, Zuccotti M (2006). Gonadotropins affect Oct-4 gene expression during mouse oocyte growth. Mol Reprod Dev.

[B40] Rosner MH, Vigano MA, Ozato K, Timmons PM, Poirier F, Rigby PW, Staudt LM (1990). A POU-domain transcription factor in early stem cells and germ cells of the mammalian embryo. Nature.

[B41] Yeom YI, Fuhrmann G, Ovitt CE, Brehm A, Ohbo K, Gross M, Hübner K, Schöler HR (1996). Germline regulatory element of Oct-4 specific for the totipotent cycle of embryonal cells. Development.

[B42] Stewart CL (2000). Oct-4, scene 1: the drama of mouse development. Nat Genet.

[B43] Boyer LA, Lee TI, Cole MF, Johnstone SE, Levine SS, Zucker JP, Guenther MG, Kumar RM, Murray HL, Jenner RG, Gifford DK, Melton DA, Jaenisch R, Young RA (2005). Core transcriptional regulatory circuitry in human embryonic stem cells. Cell.

[B44] Greco SJ, Liu K, Rameshwar P (2007). Functional similarities among genes regulated by OCT4 in human mesenchymal and embryonic stemcells. Stem Cells.

[B45] Dennis G, Sherman BT, Hosack DA, Yang J, Gao W, Lane HC, Lempicki RA (2003). DAVID: Database for Annotation, Visualization, and Integrated Discovery. Genome Biology.

[B46] Nishikimi A, Mukai J, Yamada M (1999). Nuclear translocation of nuclear factor kappa B in early 1-cell mouse embryos. Biol Reprod.

[B47] Bertazza L, Mocellin S (2008). Tumor necrosis factor (TNF) biology and cell death. Front Biosci.

[B48] Ramos SB, Stumpo DJ, Kennington EA, Phillips RS, Bock CB, Ribeiro-Neto F, Blackshear PJ (2004). The CCCH tandem zinc-finger protein Zfp36l2 is crucial for female fertility and early embryonic development. Development.

[B49] Aravind L, Koonin EV (2000). SAP – a putative DNA-binding motif involved in chromosomal organization. Trends Biochem Sci.

[B50] Nakamura T, Arai Y, Umehara H, Masuhara M, Kimura T, Taniguchi H, Sekimoto T, Ikawa M, Yoneda Y, Okabe M, Tanaka S, Shiota K, Nakano T (2007). PGC7/Stella protects against DNA demethylation in early embryogenesis. Nat Cell Biol.

[B51] Parfenov VN, Pochukalina GN, Davis DS, Reinbold R, Schöler HR, Murti KG (2003). Nuclear distribution of Oct-4 transcription factor in transcriptionally active and inactive mouse oocytes and its relation to RNA polymerase II and splicing factors. J Cell Biochem.

[B52] Kinlosh RA, Wassarman PM, Gwatkin RBL (1993). Specific gene expression during oogenesis in mice. Genes in mammalian reproduction.

[B53] Dumollard R, Duchen M, Carroll J (2007). The role of mitochondrial function in the oocyte and embryo. Curr Top Dev Biol.

[B54] Liu L, Trimarchi JR, Keefe DL (2000). Involvement of mitochondria in oxidative stress-induced cell death in mouse zygotes. Biol Reprod.

[B55] Quinn P, Harlow GM (1978). The effect of oxygen on the development of preimplantation mouse embryos in vitro. J Exp Zool.

[B56] Natsuyama S, Noda Y, Narimoto K, Umaoka Y, Mori T (1992). Release of two-cell block by reduction of protein disulfide with thioredoxin from Escherichia coli in mice. J Reprod Fertil.

[B57] Funato Y, Michiue T, Asashima M, Miki H (2006). The thioredoxin-related redox-regulating protein nucleoredoxin inhibits Wnt-beta-catenin signalling through dishevelled. Nat Cell Biol.

[B58] Imai S, Johnson FB, Marciniak RA, McVey M, Park PU, Guarente L (2000). Sir2: an NAD-dependent histone deacetylase that connects chromatin silencing, metabolism, and aging. Cold Spring Harb Symp Quant Biol.

[B59] Liu H, Colavitti R, Rovira II, Finkel T (2005). Redox-dependent transcriptional regulation. Circ Res.

[B60] Thomas FH, Ethier JF, Shimasaki S, Vanderhyden BC (2005). Follicle-stimulating hormone regulates oocyte growth by modulation of expression of oocyte and granulosa cell factors. Endocrinology.

[B61] Zuccotti M, Piccinelli A, Rossi PG, Garagna S, Redi CA (1995). Chromatin organization during mouse oocyte growth. Mol Reprod Dev.

[B62] Adjaye J, Bolton V, Monk M (1999). Developmental expression of specific genes detected in high quality cDNA libraries from single human preimplantation embryos. Gene.

[B63] Adjaye J, Huntriss J, Herwig R, BenKahla A, Brink TC, Wierling C, Hultschig C, Groth D, Yaspo ML, Picton HM, Lanzendorf SE, Gosden RG, Lehrach H (2005). Primary differentiation in the human blastocyst: Comparative molecular portraits of inner cell mass and trophectoderm cells. Stem Cells.

