# Supplementary material for: Maternal Oct-4 is a potential key regulator of the developmental competence of mouse oocytes
Source: BMC Dev Biol. 2008 Oct 6;8:97. doi: 10.1186/1471-213X-8-97 (PMC2576189; doi:10.1186/1471-213X-8-97)
Supplement: Additional file 2 — Based on their gene ontology, genes that were up-regulated or down-regulated in MIINSN oocytes were assigned to twelve biological categories most representative of their function. [file 1471-213X-8-97-S2.doc]

**Additional file 2.** Based on their gene ontology, genes that were up-regulated or down-regulated in MII^NSN^ oocytes were assigned to twelve biological categories most representative of their function.*

| Gene Ontology | | | Comparison MII^NSN^ vs. MII^SN^ | |
| --- | --- | --- | --- | --- |
|  |  |  | **Genes up-regulated in MII^NSN^ oocytes**  **(N.° of genes)** | **Genes down-regulated in MII^NSN^ oocytes**  **(N.° of genes)** |
| Transcription | |  | *1190002L16Rik****,*** *Rpo1-3****,*** (2) | ***Zfp39****, Skb1, Zhx1, Lass2, Nsd1* (5) |
|  |  | **Negative regulation of transcription** | *1500031N17Rik****,*** *Abtb1, Atf7ip, Bhlhb2****,*** *Fank1, Foxj2, Foxm1,* ***Hoxa7****,* ***Ing1****, Invs, Lsm10, Nkx6-2,* ***Nr2e1****, Rxrb, Sfrs16, Tcea3,* ***Tfam****, Tgif, Tle4, Tle6, Tmsb10, Trip4 (*22) |  |
| Protein biosynthesis | | | *1110002D22Rik,* *5330430P07Rik, Bcat1****,*** *Bzw1****,*** *Cct5, Eif3s5, Map3k12, Mark3, Mrps6, Prkg1, Prss12, Psmb2, Psmc4, Psmd7, Ptpn1, Rpl27a, Rpl36a, Rps20, Rps6, Tloc1, Uqcrc1* ***,*** *Uxt****,*** *Wbscr1* (23) | *Cpd, Mbtps2, Metap2****,*** *Mrpl3, Rpl23*(5) |
| **Cellular transport** | | | *0610040B21Rik****,*** *1110020P15Rik, 4732474A20Rik, 4833412N02Rik, Atox1, Atp6v0a1, Clta, Coq6, Grasp, Ndufv1, Rangnrf****,*** *Sec61g, Slc34a1****,*** *Slc6a9, Uqcrb, Uqcrc1* (16) | *Slc4a8****,*** *Timm17b, Cog6, Aqp1, Cope, Abcf2, Cope, Snx3****,*** *9830132G07Rik* (9) |
| **Metabolism** | | | *1110039B18Rik, Aars, Acaa2****,*** *Adprh, Alad****,*** *Aprt, Cpt2, Egfr, Eif3s1, Hadh2, Mocs2****,*** *Nsdhl, Pik3c3, Psmc5, Trpv2, Tsta3, Ubc (17)* | *Prdx6*(1) |
| **Development** | | | *Otog,* ***Myo6, Farp2,*** *Rfng,* ***Gfra1, Dnmt3l,*** *Ldb1,* ***Hoxa7****, Golga3,* ***Nr2e1****, Crkl* (11) | **Zfp39**, Dkk3 (2) |
| **Signal transduction** | | | ***Gfra1, Tsc2, Crkl****, Grasp* ***, Farp2,*** *AW742319****,*** *Iqgap1, Rab33b, Arl2* (9) | *Plcb3****,*** *1700093E07Rik, Adra2b* (3) |
| **Cell cycle** |  | |  | *Trim13, Mdm2****,*** *Cul5* (3) |
|  | **Negative regulation of cell cycle** | | *Mlh1* ***, Crkl,*** *Anapc5****, Ing1****,* ***Tsc2*** (5) |  |
| **Carbohydrate metabolism** | | | *Pfkl, Ldh1****,*** *Pfkfb3****,*** *Slc3a2, Glb1, Pgm2* (7) |  |
| **DNA metabolism** | | | *Atp6v0b, Atp5a1, Rfc4, Tk2* (4) | *Orc4l* (1) |
| **Cytoskeleton/microtubule organization** | | | *Ckap1****, Myo6****, Myo5b, Myh2* (4) | *Knsl7* (1) |
| **Apoptosis** | | | *Grim19, Bat3, Mcl1, Pdcd2* (4) |  |
| **Chromatin/nuclear organization** | | | *Terf2ip,* ***Tfam****,* ***Dnmt3l***(3) |  |
| **Various topics** | | | *Pla2g12a* (1) | *Galnt1* (1) |
| **Biological process unknown** | | | *0610033H09Rik, Aptx, Clcnkb, Fntb, Gbl, Hig1, Hn1, Mro, Paip2, Rbm14, Slco4a1, Tbrg4, Wbp2* (13) | *Sh3bgrl, Tipin, AI663987* (3) |

*: genes in bold are present in more than one biological category.
