# Supplementary material for: Maternal Oct-4 is a potential key regulator of the developmental competence of mouse oocytes
Source: BMC Dev Biol. 2008 Oct 6;8:97. doi: 10.1186/1471-213X-8-97 (PMC2576189; doi:10.1186/1471-213X-8-97)
Supplement: Additional file 3 — Additional Methods. [file 1471-213X-8-97-S3.doc]

###### Additional Methods

Antral oocytes classification and maturation to the MII stage

Each oocyte was transferred into a 5 µl droplet of M2 medium containing the fluorochrome Hoechst 33342 (50 ng/ml; Sigma-Aldrich) and incubated at 37 °C for 10 min. After staining, oocytes were classified as belonging to the SN or the NSN type, according to the presence or absence, respectively, of a ring of Hoechst-positive chromatin surrounding the nucleolus (Figures 2B and 2E show an example of the different chromatin organisation of SN and NSN antral oocytes, respectively). Classification was performed at X250 magnification on the stage of an inverted fluorescence microscope (Olympus IX70, Japan). SN and NSN oocytes were separatelly cultured for 15 hr in α-MEM medium (Sigma-Aldrich) containing 5% FBS (Invitrogen), 2 mM L-Glutamine (Invitrogen), 5 mM Taurine (Sigma-Aldrich) and 26 µg/ml sodium pyruvate in incubator at 37°C and 5% CO_2_, 90% N_2_, 5% O_2_, until they reached the MII stage.

##### RNA isolation and RT-PCR amplification

Eighteen microliters of the following reaction mixture were added to 2 µl of oocyte sample: 1 µl of exogenous *pAW109* RNA (2,500 molecules; used as internal control for normalization of data), 1X PCR buffer II, 5 mM MgCl_2_, 4 mM of each dNTP, 2.5 mM oligo d(T)16, 20 U RNase Inhibitor, 50 U MuLV reverse transcriptase (Applera, Monza, Italy). Both RT and PCR reactions were performed on an Applied Biosystems GeneAmp 9700 thermocycler. The program for the reverse transcription step was as follows: 25°C for 10 min, 42°C for 60 min, 99°C for 5 min. After the reverse transcription reaction, 5 µl of each sample were added to the following PCR reaction mix was added: 1X PCR buffer I, 200 mM of each dNTP, 250 nM of each specific primer, 1.23 IU AmpliTaq polymerase (all from Applera). The endogenous control (*Gapdh*), the exogenous control (*pAW109*), and the gene under study were coamplified during the first PCR using the following cycle program: 94°C for 5 min followed by 20 (*Zar1*, *Stella*, *Crebbp* and *Fnip1*) or 25 (*Smarca4*, *Npm2*, *Prei3*, *Jam2* and *Ogfr*) cycles at 94°C for 30 sec., 55°C for 30 sec. and 72°C for 30 sec., followed by a final elongation step at 72°C for 7 min. A second round of PCR amplification was performed separately for each gene sequence. One microliter of the first-round reaction was diluted into 24 µl of a new reaction mixture prepared as described above, with primers designed internal to the first PCR sequence (nested PCR). The second PCR cycle parameters were as follows: 94°C for 5 min followed by 20 (*Smarca4, Zar1, Npm2, Prei3* and *Stella*) or 22 (*Jam2* and *Ogfr*) or 24 (*Fnip1*) or 26 (*Crebbp*) cycles at 94°C for 30 sec., 55°C for 30 sec. and 72°C for 30 sec., followed by a final elongation step at 72°C for 7 min. The primer sequences are shown in the table below. In order to avoid genomic amplification, at least one of the external primers was chosen to overlap exon/intron boundaries. Ten microliters of PCR product were mixed with 2 µl loading buffer and were electrophoresed on a 2.5% agarose gel in 0.5% TBE containing 0.5 mg/ml ethidium bromide at 6 V/cm for 85 min. The products were visualized under short wave length UV on a Bio- Rad Gel Doc system and densitometric analysis was performed with the Bio-Rad Quantity-One software. The relative number of transcripts of the genes under study was obtained after normalization of the data with those of the exogenous control *pAW109*.

*Generation of networks and pathways with IPA*

A network is a graphical representation of the molecular relationships between genes and gene products. Genes or gene products are represented as nodes, and the biological relationship between two nodes is represented as an edge (line). All edges are supported by at least 1 reference from the literature, from a textbook, or from canonical information stored in the Ingenuity Pathways Knowledge Base. Human, mouse, and rat orthologs of a gene are stored as separate objects in the Ingenuity Pathways Knowledge Base, but are represented as a single node in the network. The intensity of the node color indicates the degree of up- (red) or down- (green) regulation. Nodes are displayed using various shapes that represent the functional class of the gene product. Edges are displayed with various labels that describe the nature of the relationship between the nodes (e.g., P for phosphorylation, T for transcription).

After data uploading, each gene identifier was mapped to its corresponding gene object in the Ingenuity Pathways Knowledge Base. A *fold-change* cutoff of 1.5 was set to identify genes whose expression was significantly differentially regulated. These genes, called focus genes, were overlaid onto a global molecular network developed from information contained in the Ingenuity Pathways Knowledge Base. Networks of these focus genes were then algorithmically generated based on their connectivity.

The Functional Analysis of a network identified the biological functions that were most significant to the genes in the network. The network genes associated with biological functions in the Ingenuity Pathways Knowledge Base were considered for the analysis. Fischer’s exact test was used to calculate a p-value determining the probability that each biological function and/or disease assigned to that network is due to chance alone. The used score is computed as the base 10 logarithm of the p-value.

Canonical pathways analysis identified the pathways from the Ingenuity Pathways Analysis library of canonical pathways that were most significant to the data set. Genes from the data set that met the p-value cut-off of 0.05 and were associated with a canonical pathway in the Ingenuity Pathways Knowledge Base were considered for the analysis. The significance of the association between the data set and the canonical pathway was assessed through Fischer’s exact test. The resulting p-value determines the probability that the association between the genes in the dataset and the canonical pathway is explained by chance alone.
