# Supplementary figures and images for: Maternal Oct-4 is a potential key regulator of the developmental competence of mouse oocytes
Source: BMC Dev Biol. 2008 Oct 6;8:97. doi: 10.1186/1471-213X-8-97 (PMC2576189; doi:10.1186/1471-213X-8-97)

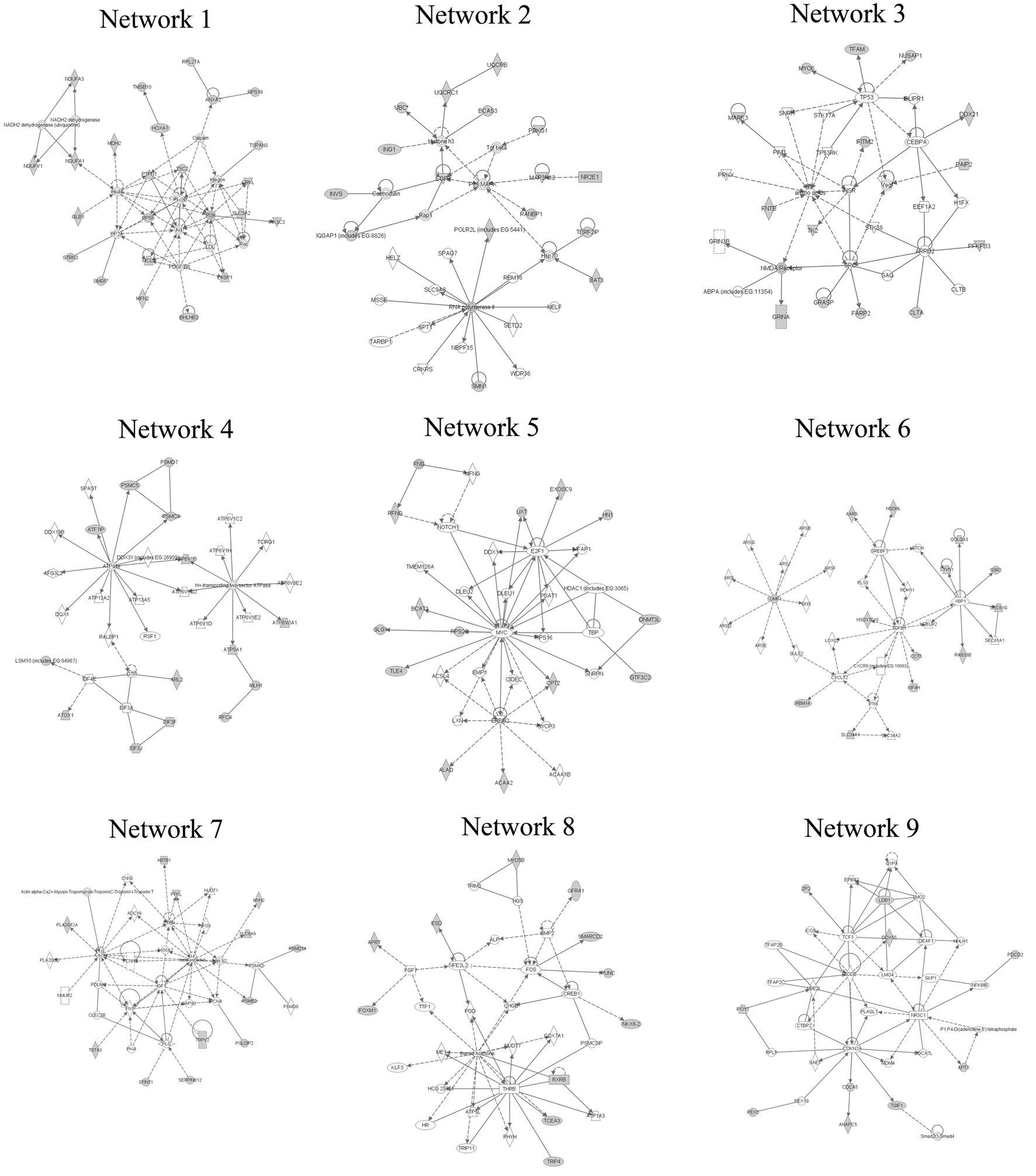

Supplement: Additional file 4 — Top nine gene expression networks generated by IPA with the list of genes up-regulated in MIINSN oocytes. Grey symbols are focus genes (use the zoom in tool to enlarge the networks). See Additional file 6 for the symbols legend. [file 1471-213X-8-97-S4.tiff]

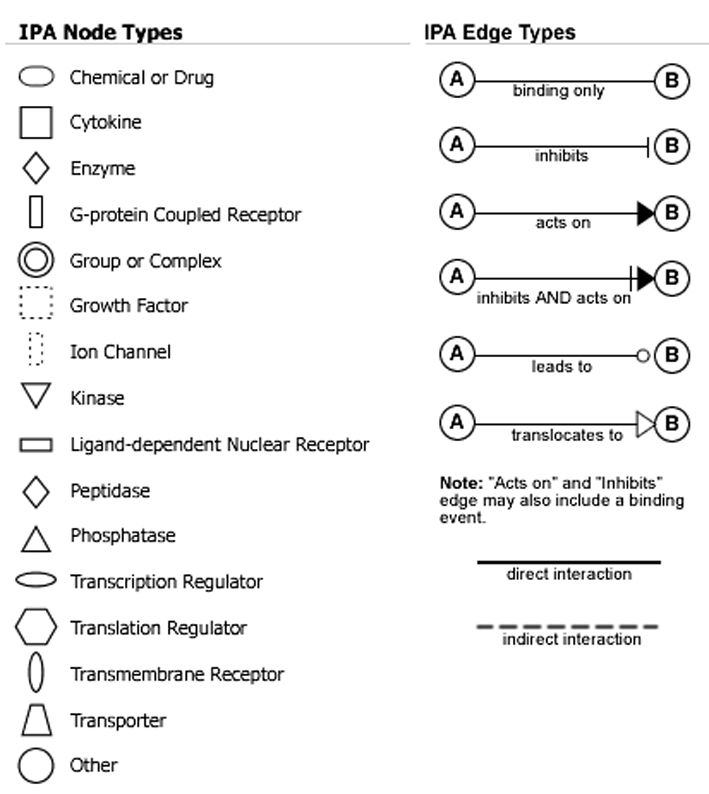

Supplement: Additional file 6 — Legend of symbols used in the figures shown in Additional files 4, 7, 10 and 13. [file 1471-213X-8-97-S6.tiff]

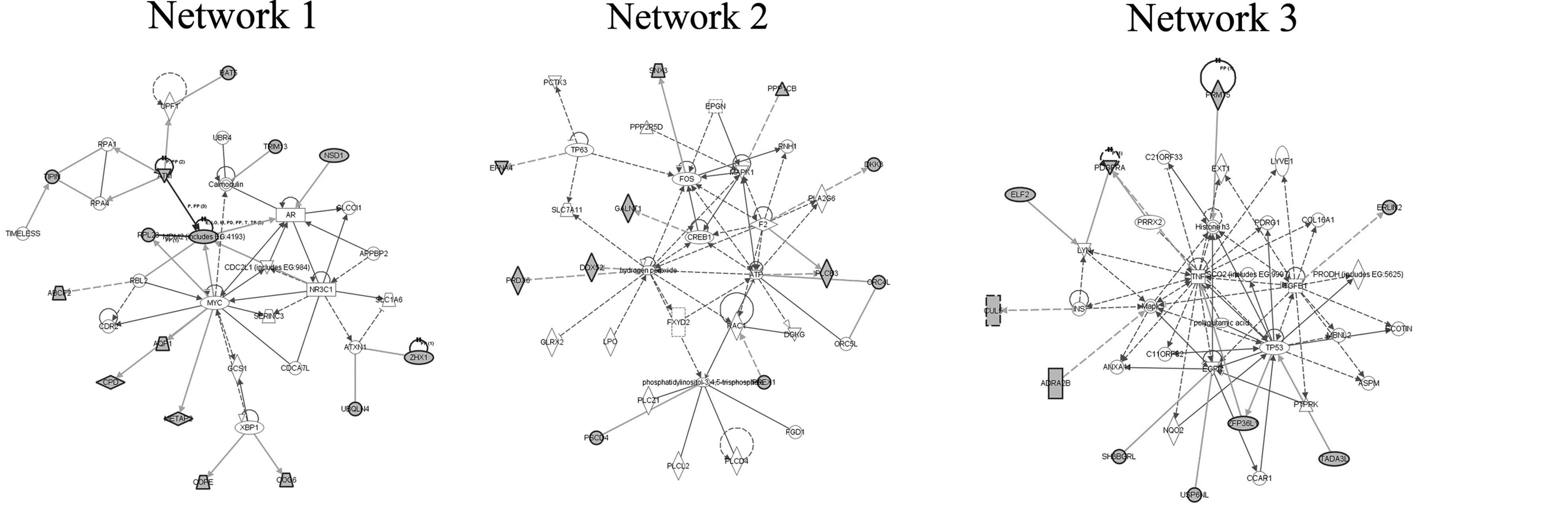

Supplement: Additional file 7 — Top three gene expression networks generated by IPA with the list of genes down-regulated in MIINSN oocytes. Grey symbols are focus genes (use the zoom in tool to enlarge the networks). [file 1471-213X-8-97-S7.tiff]

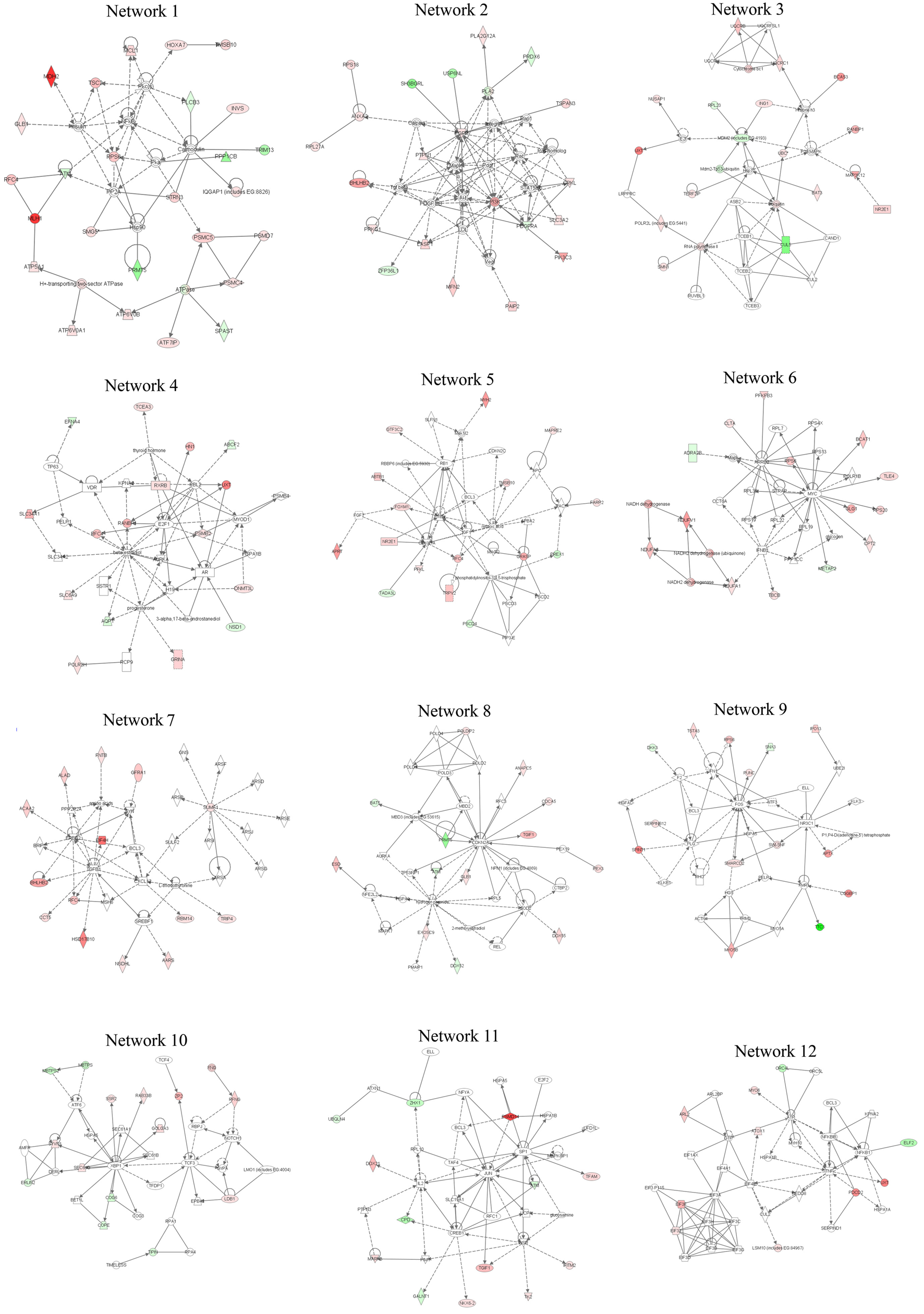

Supplement: Additional file 10 — Top twelve gene expression networks generated by IPA with the list of genes regulated in MIINSN oocytes. Green symbols, genes up-regulated; red symbols, genes down-regulated (the intensity of the colour indicates the level of regulation) (use the zoom in tool to enlarge the networks). [file 1471-213X-8-97-S10.tiff]

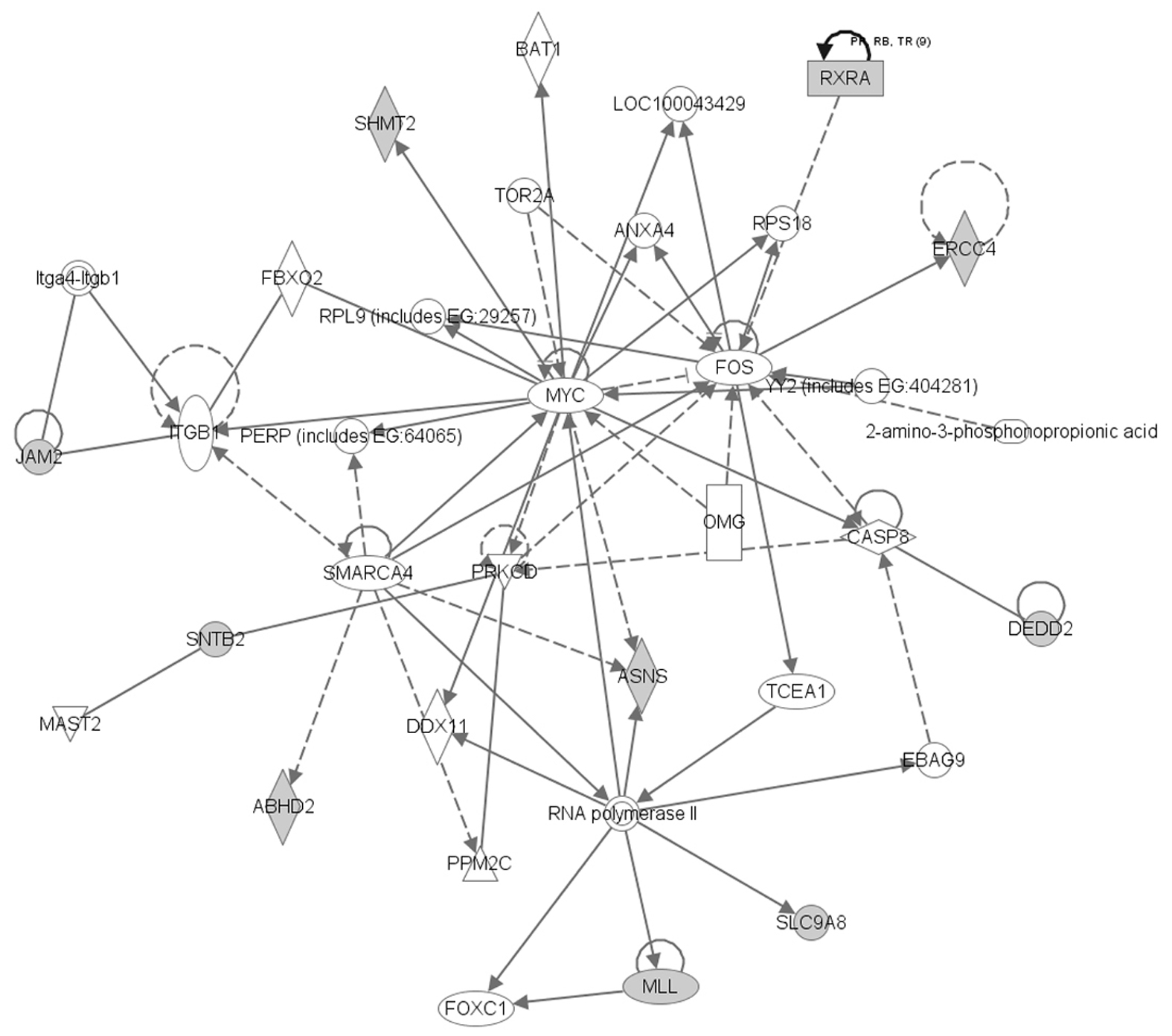

Supplement: Additional file 13 — Gene expression network 1 generated by IPA with the list of genes expressed solely in MIINSN oocytes. Grey symbols are focus genes (use the zoom in tool to enlarge the networks). [file 1471-213X-8-97-S13.tiff]

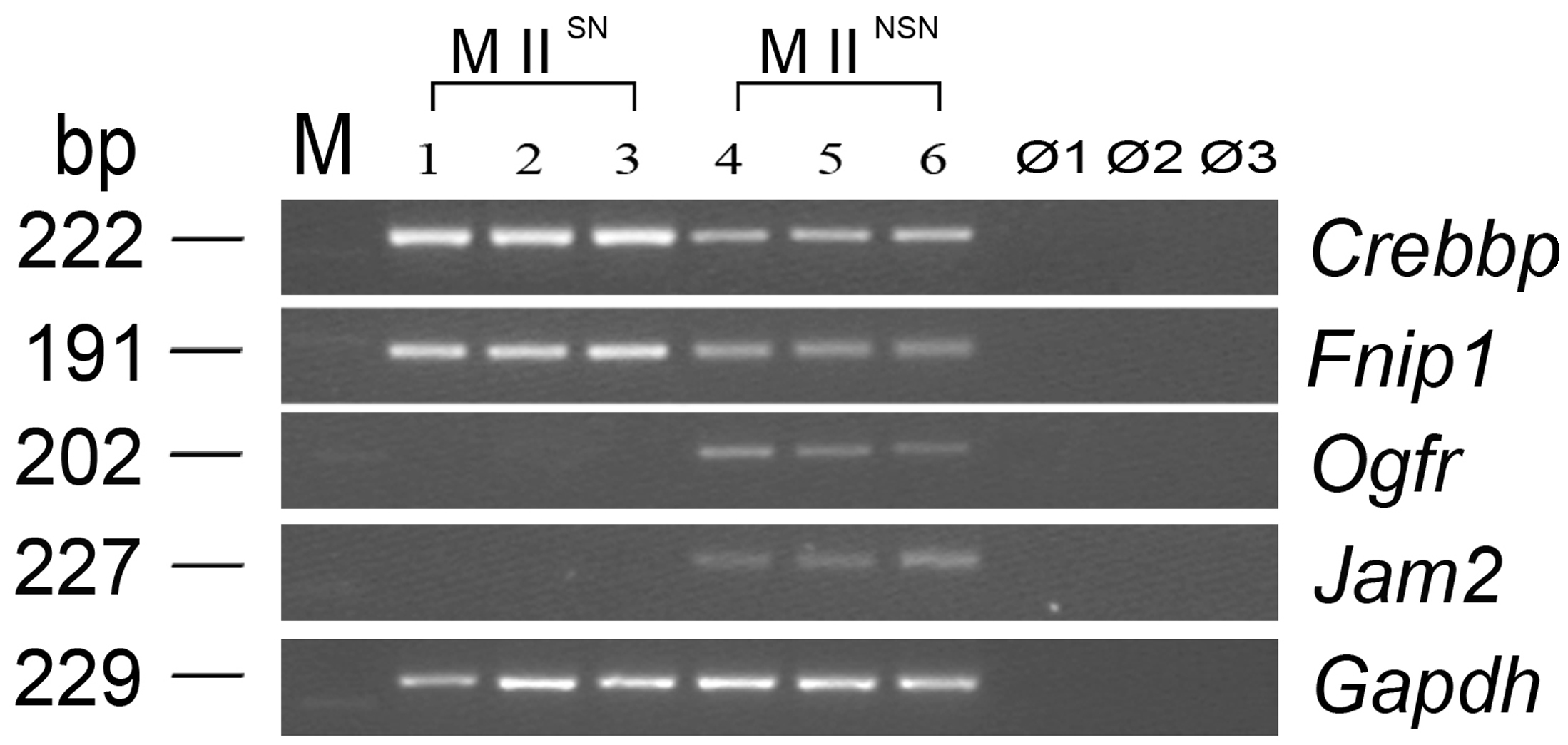

Supplement: Additional file 14 — The gel electrophoresis shows the product of amplification of two genes expressed solely in MIINSN oocytes (Ogfr and Jam2) and two genes expressed more abundantly in MIISN oocytes (Crebbp and Fnip1). Gapdh, endogenous control whose transcripts are present equally in the two types of oocytes; 1–3, three different single MIINN oocytes; 4–6, three different single MIINSN oocytes. ∅1, RT blank; ∅2, first PCR blank; ∅3, second PCR blank; M, low mass ladder marker. [file 1471-213X-8-97-S14.tiff]
