# Supplementary material for: Maternal Oct-4 is a potential key regulator of the developmental competence of mouse oocytes
Source: BMC Dev Biol. 2008 Oct 6;8:97. doi: 10.1186/1471-213X-8-97 (PMC2576189; doi:10.1186/1471-213X-8-97)
Supplement: Additional file 5 — Networks generated by IPA for focus genes that are down-regulated in MIINSN oocytes when compared to MIISN oocytes. [file 1471-213X-8-97-S5.doc]

**Additional file 5.** Networks generated by IPA for focus genes that are down-regulated in MII^NSN^ oocytes when compared to MII^SN^ oocytes.

| **Network** | **Genes in Network** | **Score** | **Focus Genes** | Top Functions |
| --- | --- | --- | --- | --- |
| 1 | Abcf2, Appbp2, Aqp1, Ar, Atm, Atxn1, Bat5, Calmodulin, Cdc2l1 (Includes Eg:984), Cdca7l, Cdr2, Cog6, Cope, Cpd, Gcs1, Glcci1, Mdm2 (Includes Eg:4193), Metap2, Myc, Nr3c1, Nsd1, Rbl2, Rpa1, Rpa4, Rpl23, Serinc3, Slc1a6, Timeless, Tipin, Trim13, Ubqln4, Ubr4, Upf1, Xbp1, Zhx1 | 33 | 15 | Gene Expression, Cancer, Cell Cycle |
| 2 | *Atp, Creb1, Ddx52, Dgkg, Dkk3, Efna4, Epgn, F2, Fgd1, Fos, Fxyd2, Galnt1, Glrx2, Hydrogen Peroxide, Lpo, Mapk1, Orc4l, Orc5l, Pctk3, Phosphatidylinositol-3,4,5-Trisphosphate, Pla2g6, Plcb3, Plcd4, Plcl2, Plcz1, Ppp1cb, Ppp2r5d, Prdx6, Prex1, Pscd4, Rac1, Rnh1, Slc7a11, Snx3, Tp63* | 22 | 11 | Cell Death, Hematological System Development and Function, Tissue Development |
| 3 | *Adra2b, Anxa11, Aspm, C11orf82, C21orf33, Ccar1, Col16a1, Cul5, Egfr, Elf2, Erlin2, Ext1, Histone H3, Ins, Lyn, Lyve1, Mapk, Mbnl2, Nqo2, Pdgfra, Pdrg1, Polyglutamic Acid, Prmt5, Prodh (Includes Eg:5625), Prrx2, Ptprk, Sco2 (Includes Eg:9997), Scotin, Sh3bgrl, Tada3l, Tgfb1, Tnf, Tp53, Usp6nl, Zfp36l1* | 20 | 10 | Cancer, Cell Death, Renal and Urological Disease |
| 4 | *Kif15, Mki67, Tpx2* | 2 | 1 | Cell Cycle, Embryonic Development, Hair and Skin Development and Function |
| 5 | *Fmr1, Tal1, Ttc3* | 2 | 1 | Cardiovascular System Development and Function, Connective Tissue Development and Function, Developmental Disorder |
| 6 | *Atf6, Mbtps, Mbtps2* | 2 | 1 | Gene Expression, Cancer, Cell Death |
| 7 | *Atpase, Spast, Spg3a* | 2 | 1 | Genetic Disorder, Neurological Disease, Cancer |
