# Supplementary material for: Maternal Oct-4 is a potential key regulator of the developmental competence of mouse oocytes
Source: BMC Dev Biol. 2008 Oct 6;8:97. doi: 10.1186/1471-213X-8-97 (PMC2576189; doi:10.1186/1471-213X-8-97)
Supplement: Additional file 8 — Pathways generated by IPA for focus genes that are down-regulated in MIINSN oocytes. [file 1471-213X-8-97-S8.doc]

**Additional file 8.** Pathways generated by IPA for focus genes that are down-regulated in MII^NSN^ oocytes.

| Pathway | **Gene Symbol** | Description | **Network** | **Location** | **Type** |
| --- | --- | --- | --- | --- | --- |
| Cell cycle: G2/M DNA damage checkpoint regulation | Atm | ataxia telangiectasia mutated | 1 | Nucleus | kinase |
|  | Mdm2 | Mdm2, transformed 3T3 cell double minute 2, p53 binding protein (mouse) | 1 | Nucleus | transcription regulator |
| Hypoxia Signalling | Atm | ataxia telangiectasia mutated | 1 | Nucleus | kinase |
|  | Mdm2 | Mdm2, transformed 3T3 cell double minute 2, p53 binding protein (mouse) | 1 | Nucleus | transcription regulator |
| p53 signalling | Atm | ataxia telangiectasia mutated | 1 | Nucleus | kinase |
|  | Mdm2 | Mdm2, transformed 3T3 cell double minute 2, p53 binding protein (mouse) | 1 | Nucleus | transcription regulator |
| Chemokine Signalling | Plcb3 | phospholipase C, beta 3 (phosphatidylinositol-specific) | 2 | Cytoplasm | enzyme |
|  | Ppp1cb | protein phosphatase 1, catalytic subunit, beta isoform | 2 | Cytoplasm | phosphatase |
| Phospholipid degradation | Plcb3 | phospholipase C, beta 3 (phosphatidylinositol-specific) | 2 | Cytoplasm | enzyme |
|  | Prdx6 | peroxiredoxin 6 | 2 | Cytoplasm | enzyme |
| Synaptic long term potentiation | Plcb3 | phospholipase C, beta 3 (phosphatidylinositol-specific) | 2 | Cytoplasm | enzyme |
|  | Ppp1cb | protein phosphatase 1, catalytic subunit, beta isoform | 2 | Cytoplasm | phosphatase |
| Methane Metabolism | Prdx6 | peroxiredoxin 6 | 2 | Cytoplasm | enzyme |
| Glycerophospholipid Metabolism | Plcb3 | phospholipase C, beta 3 (phosphatidylinositol-specific) | 2 | Cytoplasm | enzyme |
|  | Prdx6 | peroxiredoxin 6 | 2 | Cytoplasm | enzyme |
| Aminophosphonate metabolism | Skb1 | protein arginine methyltransferase 5 | 3 | Cytoplasm | enzyme |
| Endoplasmic Reticulum Stress Pathway | Mbtps2 | membrane-bound transcription factor peptidase, site 2 | 6 | Cytoplasm | peptidase |
