# Supplementary material for: Maternal Oct-4 is a potential key regulator of the developmental competence of mouse oocytes
Source: BMC Dev Biol. 2008 Oct 6;8:97. doi: 10.1186/1471-213X-8-97 (PMC2576189; doi:10.1186/1471-213X-8-97)
Supplement: Additional file 9 — Networks generated by IPA for all the focus genes that are regulated in MIINSN oocytes when compared to MIISN oocytes. [file 1471-213X-8-97-S9.doc]

**Additional file 9.** Networks generated by IPA for all the focus genes that are regulated in MII^NSN^ oocytes when compared to MII^SN^ oocytes.

| **Network** | Genes in network | **Score** | **Focus genes** | **Top Functions** |
| --- | --- | --- | --- | --- |
| 1 | Atf7ip, Atm, Atp5a1, Atp6v0a1, Atp6v0b, Atpase, Calmodulin, Glb1, H+-Transporting Two-Sector Atpase, Hoxa7, Hsp90, Insulin, Invs, Iqgap1 (Includes Eg:8826), Mcl1, Mdh2, Mlh1, Nfkb, Pka, Pkc(S), Plcb3, Pp2a, Ppp1cb, Prmt5, Psmc4, Psmc5, Psmd7, Rfc4, Rps6, Smg5, Spast, Strn3, Tmsb10, Trim13, Tsc2 | 47 | 26 | Cell Cycle, Cancer, Cellular Compromise |
| 2 | *Akt, Anxa2, Bhlhb2, Calpain, Crkl, Egfr, Integrin, Lasp1, Ldl, Mapk, Mfn2, Paip2, Pdgf, Pdgf Bb, Pdgfra, Pi3k, Pik3c3, Pla2, Pla2g12a, Prdx6, Prkg1, Ptpn1, Rac, Rap1, Ras Homolog, Rpl27a, Rps18, Sh3bgrl, Slc3a2, Stat5a/B, Tgf Beta, Tspan3, Usp6nl, Vegf, Zfp36l1* | 33 | 20 | Cellular Movement, Connective Tissue Development and Function, Cellular Growth and Proliferation |
| 3 | *Asb2, Bat3, Bcas3, Cand1, Cul2, Cul5, Cytochrome Bc1, E2f, Histone H3, Hsp70, Ing1, Lrpprc, Map3k12, Mdm2 (Includes Eg:4193), Mdm2-Tp53-Ubiquitin, Nr2e1, Nusap1, P38 Mapk, Polr2l (Includes Eg:5441), Ranbp1, Rna Polymerase Ii, Rpl23, Ruvbl1, Smn1, Tceb1, Tceb2, Tceb3, Terf2ip, Ubc, Ubiquitin, Uqcrb, Uqcrc1, Uqcrfsl1, Uqcrh, Uxt* | 26 | 17 | Post-Translational Modification, Cellular Function and Maintenance, Cancer |
| 4 | *3-Alpha,17-Beta-Androstanediol, Abcf2, Aqp1, Ar, Aurka, Beta-Estradiol, Dnmt3l, E2f1, Efna4, Grina, H19, Hn1, Hspa1b, Kpna2, Myod1, Nsd1, Pelp1, Polr3h, Progesterone, Psmb2, Psmb4, Ranbp1, Rbl2, Rcp9, Rfc4, Rxrb, Slc34a1, Slc34a2, Slc6a9, Sstr1, Tcea3, Thyroid Hormone, Tp63, Uxt, Vdr* | 24 | 16 | Gene Expression, Cell Cycle, Cancer |
| 5 | *Abtb1, Apba2, Apc, Aprt, Bcl3, Cdkn1a, Cdkn2c, Farp2, Fgf7, Foxm1, Grasp, Gtf3c2, Igf1, Magi2, Mapre2, Mek1/2, Myh2, Nr2e1, Pfkl, Phosphatidylinositol-3,4,5-Trisphosphate, Pip3-E, Prex1, Pscd2, Pscd3, Pscd4, Pten, Rac1, Rb1, Rbbp6 (Includes Eg:5930), Retinoic Acid, Rfc4, Slfn1, Tada3l, Tmsb10, Trpv2* | 23 | 16 | Cancer, Cellular Growth and Proliferation, Gastrointestinal Disease |
| 6 | *Adra2b, Arrb2, Bcat1, Cct6a, Clta, Cpt2, Glg1, Glycogen, Ifnb1, Mapk, Metap2, Myc, Nadh Dehydrogenase, Nadh2 Dehydrogenase, Nadh2 Dehydrogenase (Ubiquinone), Ndufa1, Ndufa3, Ndufv1, Pfkfb3, Polr1b, Ppp1cc, Rpl7, Rpl19, Rpl22, Rpl35, Rps6, Rps13, Rps19, Rps20, Rps4x, Strap, Tbcb, Tle4* | 20 | 14 | Protein Synthesis, Cancer, Cell Cycle |
| 7 | *Aars, Acaa2, Alad, Amino Acids, Arsa, Arsb, Arsd, Arse, Arsf, Arsg, Arsi, Arsj, Bcl3, Bhlhb2, Brip1, Cct5, Cxcl12, Eif4h, Erbb2, Fntb, Fyn, Gfra1, Gns, Hsd17b10, L-Triiodothyronine, Msh6, Nsdhl, Ppp2r2a, Rbm14, Rfc4, Srebf1, Sulf2, Sumf1, Tgfb1, Trip4* | 20 | 14 | Cell Morphology, Cellular Assembly and Organization, Nervous System Development and Function |
| 8 | *2-Methoxyestradiol, Anapc5, Atm, Aurka, Bat5, Cdca5, Cdkn2a, Ctbp2, Ddx52, Ddx55, Esd, Exosc9, Glb1, Hspa9, Hydrogen Peroxide, Mapk7, Mbd2, Mbd3 (Includes Eg:53615), Nfe2l2, Npm1 (Includes Eg:4869), Pex3, Pex19, Pmaip1, Pold1, Pold2, Pold3, Pold4, Poldip2, Prmt5, Rel, Rfc3, Rpl5, Sod2, Tgif1, Tp53inp1* | 18 | 13 | Cell Cycle, Cancer, Cell Death |
| 9 | *Actn4, Aptx, Bcl3, Cggbp1, Dkk3, Ell, F2, Fmr1, Fn1, Fos, Hgfac, Hgs, Hspa5, Ipo13, Klk3, Klkb1, Myo5a, Myo5b, Nr3c1, Ntf3, P1,P4-Di(Adenosine-5') Tetraphosphate, Pelp1, Plg, Punc, Rho, Rps6, Serpinb12, Smarcd2, Snx3, Spint1, Swi-Snf, Trim3, Tsta3, Ttc3, Ube2i* | 18 | 13 | Protein Synthesis, Gene Expression, Cell-To-Cell Signaling and Interaction |
| 10 | *Amfr, Atf6, Bet1l, Cog3, Cog6, Cope, Derl1, Epb42, Erlin2, Fng, Golga3, Gypa, Hspa5, Ldb1, Lmo1 (Includes Eg:4004), Mbtps, Mbtps2, Notch1, Rab33b, Rbpj, Rfng, Rpa1, Rpa4, Sec61a1, Sec61b, Sec61g, Ssr2, Syvn1, Tcf3, Tcf4, Tfdp1, Timeless, Tipin, Xbp1, Zp2* | 18 | 13 | Gene Expression, Cellular Function and Maintenance, DNA Replication, Recombination, and Repair |
| 11 | *Atm, Atxn1, Bcl3, Cpd, Creb1, Ddx21, E2f2, Ell, Galnt1, Glucosamine, Hspa5, Hspa1b, Ifitm2, Il2, Insr, Jun, Mapk8ip1, Mark3, Nfya, Nkx6-2, Pim1, Psmd14, Ptpn3, Rfc1, Rpl10, Slc19a1, Sp1, Taf4, Tfam, Tgif1, Tk2, Ubqln4, Ucp1, Ufd1l, Zhx1* | 18 | 13 | Cell Death, Gene Expression, Neurological Disease |
| 12 | *Arl2, Arl2bp, Atox1, Atp, Bcl3, Cul2, Eif1ax, Eif3 P115, Eif3a, Eif3b, Eif3c, Eif3d, Eif3f, Eif3g, Eif3h, Eif3j, Eif4a1, Eif4e, Elf2, Gtp, Hspa1a, Hspa1b, Kpna2, Lsm10 (Includes Eg:84967), Myh10, Myo6, Nedd8, Nfkb1, Nfkbib, Orc4l, Orc5l, Pdcd2, Serpind1, Tnf, Uxt* | 12 | 10 | Protein Synthesis, Gene Expression, RNA Trafficking |
