# Supplementary material for: Maternal Oct-4 is a potential key regulator of the developmental competence of mouse oocytes
Source: BMC Dev Biol. 2008 Oct 6;8:97. doi: 10.1186/1471-213X-8-97 (PMC2576189; doi:10.1186/1471-213X-8-97)
Supplement: Additional file 11 — Pathways generated by IPA for focus genes that are regulated in MIINSN oocytes. [file 1471-213X-8-97-S11.doc]

**Additional file 11.** Pathways generated by IPA for focus genes that are regulated in MII^NSN^ oocytes.

| **Pathway** | **Gene** | **Network** | **Location** | Type |
| --- | --- | --- | --- | --- |
| Protein Ubiquitination | Psmc4 | 1 | Nucleus | peptidase |
|  | *Psmc5* | 1 | Nucleus | transcription regulator |
|  | *Psmd7* | 1 | Cytoplasm | other |
| Oxidative Phosphorylation | *Atp5a1* | 1 | Cytoplasm | transporter |
|  | *Atp6v0a1* | 1 | Cytoplasm | transporter |
|  | *Atp6v0b* | 1 | Cytoplasm | transporter |
| Ubiquinone Biosynthesis | *Prmt5* | 1 | Cytoplasm | enzyme |
| Cell Cycle: G2/M DNA Damage Checkpoint Regulation | *Atm* | 1 | Nucleus | kinase |
| Protein Ubiquitination | *Mdm2* | 3 | Nucleus | transcription regulator |
|  | *Ubc* | 3 | Cytoplasm | other |
| Oxidative Phosphorylation | *Uqcrb* | 3 | Cytoplasm | enzyme |
|  | *Uqcrc1* | 3 | Cytoplasm | enzyme |
| Cell Cycle: G2/M DNA Damage Checkpoint Regulation | *Mdm2* | 3 | Nucleus | transcription regulator |
|  | *Ubc* | 3 | Cytoplasm | other |
| Oxidative Phosphorylation | *Ndufa1* | 6 | Cytoplasm | enzyme |
|  | *Ndufa3* | 6 | Cytoplasm | enzyme |
|  | *Ndufv1* | 6 | Cytoplasm | enzyme |
| Fructose and Mannose Metabolism | *Pfkfb3* | 6 | Cytoplasm | kinase |
| Ubiquinone Biosynthesis | *Ndufa1* | 6 | Cytoplasm | enzyme |
|  | *Ndufa3* | 6 | Cytoplasm | enzyme |
|  | *Ndufv1* | 6 | Cytoplasm | enzyme |
| Valine, Leucine and Isoleucine Degradation | *Bcat1* | 6 | Cytoplasm | enzyme |
