# Supplementary material for: Maternal Oct-4 is a potential key regulator of the developmental competence of mouse oocytes
Source: BMC Dev Biol. 2008 Oct 6;8:97. doi: 10.1186/1471-213X-8-97 (PMC2576189; doi:10.1186/1471-213X-8-97)
Supplement: Additional file 12 — Networks generated by IPA for focus genes that are expressed exclusively MIINSN and not in MIISN oocytes. [file 1471-213X-8-97-S12.doc]

**Additional file 12 .** Networks generated by IPA for focus genes that are expressed exclusively MII^NSN^ and not in MII^SN^ oocytes.*

| **Network** | **Genes in network** | **Score** | **Focus genes** | **Top functions** |
| --- | --- | --- | --- | --- |
| 1 | 2-Amino-3-Phosphonopropionic Acid, **Abhd2**, Anxa4, **Asns**, Bat1, Casp8, Ddx11, **Dedd2**, Ebag9, **Ercc4**, Fbxo2, Fos, Foxc1, Itga4-Itgb1, Itgb1, Jam2, Loc100043429, Mast2, **Mll**, Myc, Omg, Perp (Includes Eg:64065), Ppm2c, Prkcd, Rna Polymerase Ii, Rpl9 (Includes Eg:29257), Rps18, **Rxra**, **Shmt2**, **Slc9a8**, Smarca4, **Sntb2**, Tcea1, Tor2a, Yy2 (Includes Eg:404281) | 26 | 10 | Hair and Skin Development and Function, Organ Morphology, Cellular Development |
| 2 | *Nfyb,* ***Pxmp4*** | 3 | 1 | Gene Expression |
| 3 | *Ank1,* ***Obscn*** *(Includes Eg:84033), Ttn* | 3 | 1 | Cell Morphology, Cellular Assembly and Organization, Cellular Development |
| 4 | *Lama5,* ***Ogfr****, Penk* | 3 | 1 | Renal and Urological System Development and Function, Cancer, Cardiovascular Disease |

*: highlighted in bold are the MII^NSN^-specific genes.
